# Comparative transcriptome and metabolome analyses of two strawberry cultivars with different storability

**DOI:** 10.1371/journal.pone.0242556

**Published:** 2020-12-02

**Authors:** Kyeonglim Min, Gibum Yi, Jeong Gu Lee, Hyun Sook Kim, Yoonpyo Hong, Jeong Hee Choi, Sooyeon Lim, Eun Jin Lee

**Affiliations:** 1 Department of Plant Science, College of Agriculture and Life Sciences, Seoul National University, Seoul, Republic of Korea; 2 Plant Genomics and Breeding Institute, Seoul National University, Seoul, Republic of Korea; 3 Strawberry Research Institute, Chungnam ARES, Nonsan, Republic of Korea; 4 National Institute of Horticultural and Herbal Science, Rural Development Administration, Wanju-gun, Republic of Korea; 5 Korea Food Research Institute, Wanju-gun, Jeollabuk-do, Republic of Korea; 6 Research Institute of Agriculture and Life Sciences, Seoul National University, Seoul, Republic of Korea; Università degli Studi di Pavia, ITALY

## Abstract

Postharvest storability is an important trait for breeding strawberry (*Fragaria* × *ananassa* Duch.). We evaluated the postharvest fruit quality of five strawberry cultivars (‘Durihyang’, ‘Kingsberry’, ‘Maehyang’, ‘Seolhyang’, and ‘Sunnyberry’) and identified differences in their fruit ripening during the transition from the big-green to fully-red stage between two cultivars with the highest (‘Sunnyberry’) and lowest (‘Kingsberry’) storability, using comparative transcriptome and -metabolome analysis. The differentially expressed genes revealed transcriptome changes related to anthocyanin biosynthesis and cell walls. Consistently, the metabolites of both cultivars showed general changes during ripening along with cultivar-specific characteristics in sugar and amino acid profiles. To identify the genes responsible for storability differences, we surveyed the expression of transcription factors, and found that the expression levels of *WRKY31*, *WRKY70*, and *NAC83* correlated with delayed senescence and increased storability. Among them, the expression levels of *NAC83*, and its downstream target genes, in the five cultivars suggested that *NAC83* expression can be used to predict postharvest strawberry fruit storability.

## Introduction

Strawberry (*Fragaria* × *ananassa* Duch.) fruit is an economically important horticultural crop, the cultivation and consumption of which has significantly increased worldwide since the 1960s. Production and exportation increased over three-fold in the last two decades [1], and overall production reached 9 million metric tons in 2016, with >10% of the strawberry fruits being exported. Strawberry fruit is characterized by a sweet flavor, juicy texture, and rich nutritive value. Strawberry fruit contains > 300 volatile compounds, including acids, alcohols, aldehydes, ketones, and terpenes [2], and various natural antioxidants, such as anthocyanins and vitamins [3]. However, strawberry fruit readily becomes senescent after harvest and has a short shelf-life due to its highly sensitive response to biotic and abiotic stresses [4].

Postharvest senescence is a major cause of decreased strawberry fruit quality and storability, and is accompanied by physiological and biochemical changes during fruit ripening. Accumulation of cell wall-degrading enzymes (including pectinesterase, pectin-methylesterase, and polygalacturonase) decrease fruit firmness and neutral-sugar content in cell walls (such as xylose, galactose, and arabinose) during strawberry fruit ripening [4,5]. Moreover, they degrade the middle lamella, which accelerates fruit softening and changes the fruit texture [6,7]. Plant hormones can also control senescence. Unlike climacteric fruit ripening, which is affected by ethylene, abscisic acid (ABA) is reportedly associated with ripening of non-climacteric fruit such as strawberries [8]. A mutant of the 9-cis epoxycarotenoid dioxygenase (*NCED*) gene, which encodes an enzyme that cleaves 9-cis xanthophylls to xanthoxin (an ABA precursor), caused decreased ABA levels and results in un-colored strawberry fruit [9]. ABA regulation of fruit ripening is associated with sucrose. Sucrose transporter-overexpressing lines have been shown to increase sucrose and ABA levels and accelerate strawberry fruit ripening [10]. Additionally, sucrose and ABA co-regulate fruit ripening by affecting expressions of ABA and sucrose signaling genes and ripening-related genes [11].

Plant senescence is genetically regulated by various transcription factors (TFs) [12–14]. The NAC (NAM, ATAF1/2, and CUC1/2), WRKY, AP2 (APETALA2)/ethylene-responsive factor (ERF), and MYB proteins were characterized as TFs of leaf or fruit senescence in *Arabidopsis thaliana* [15,16]. Several TFs, including NAC, AP2/ERF, MYB, and WRKY, were differentially expressed in different citrus (*Citrus* spp.) varieties during postharvest storage, revealing their potential regulatory roles [17]. Several *NAC* and *MYB* genes were also associated with ripening and senescence control in strawberry fruit [18,19]. Additionally, NAC proteins identified in banana fruit regulate ripening by interacting with ethylene insensitive 3-like protein 5, which participates in ethylene signaling [20]. Ripening and senescence are continuous processes controlled by complex networks; therefore, investigating the roles of TFs capable of regulating multiple factors in these networks, may prove beneficial for understanding and controlling these processes.

Fruit storability largely depends on the genetic background, and efforts to develop sweet and soft, yet highly storable cultivars, have been prioritized in strawberry breeding [21]. Sequencing the strawberry genome enabled genetic dissection for improving postharvest storability [22]. Transcriptomic approaches have been performed to better understand the key molecular biological processes, such as ripening [23,24]. In fact, Yuan et al. reported a tissue-specific transcriptome of strawberry fruit [25]. These studies demonstrate how the transcriptome is regulated temporally and spatially during ripening.

Comprehensive investigations including both transcriptome and metabolome data provides a powerful means for understanding fruit ripening and storability at both the molecular and physiological levels. Using such strategies, TFs, such as *AP2a* and *SlERF6* have been shown to be involved in tomato fruit ripening [26,27]. Similar approaches have been applied to understand molecular and biochemical changes during ripening of black raspberry fruit [28], tomato, pepper [29], and strawberry fruit [30]. However, such integrative approaches remain to be investigated in strawberry fruit ripening and storability.

In recent years, efforts to understand genetic regulation of strawberry fruit ripening have been made. Transcriptome and degradome analyses showed TFs belonging to WRKY and heat shock factor families and several miRNAs may play roles in regulation of the ripening [30]. An atypical HLH (*FaPRE1*) belonging to the basic helix-loop-helix/helix-loop-helix (bHLH/HLH) family was identified as a ripening-related TF in strawberry fruit [31,32]. Moreover, *FvTCP9* (TEOSINTE BRANCHED 1, CYCLOIDEA, and PROLIFERATING4 CELL FACTORS) was revealed as the TF related to fruit ripening via regulation of biosynthesis of ABA and anthocyanin in woodland strawberry (*Fragaria vesca*) fruit [33]. However, the link between genetic regulation and fruit storability remains poorly understood. To breed a strawberry cultivar with enhanced fruit storability, it is important to identify the molecular mechanism associated with fruit senescence.

Here, we hypothesized that variations in the storability of strawberry cultivars are due to differences in TFs. To test this hypothesis, we compared the postharvest fruit storability of five Korean strawberry cultivars and selected two with the highest and lowest storability for RNA sequencing (RNA-Seq) and metabolite analysis. By comparing the transcriptomes and metabolomes at two stages during fruit ripening, we selected candidate genes that may be important TFs of storability. We also tested the applicability of the candidate genes for predicting storability with all five cultivars. Our study provides information regarding the genetic regulation of strawberry fruit ripening and senescence. Furthermore, our findings provide a theoretical basis for selecting and breeding strawberry cultivars with enhanced fruit storability.

## Materials and methods

### Plant materials

**Fig 1:**
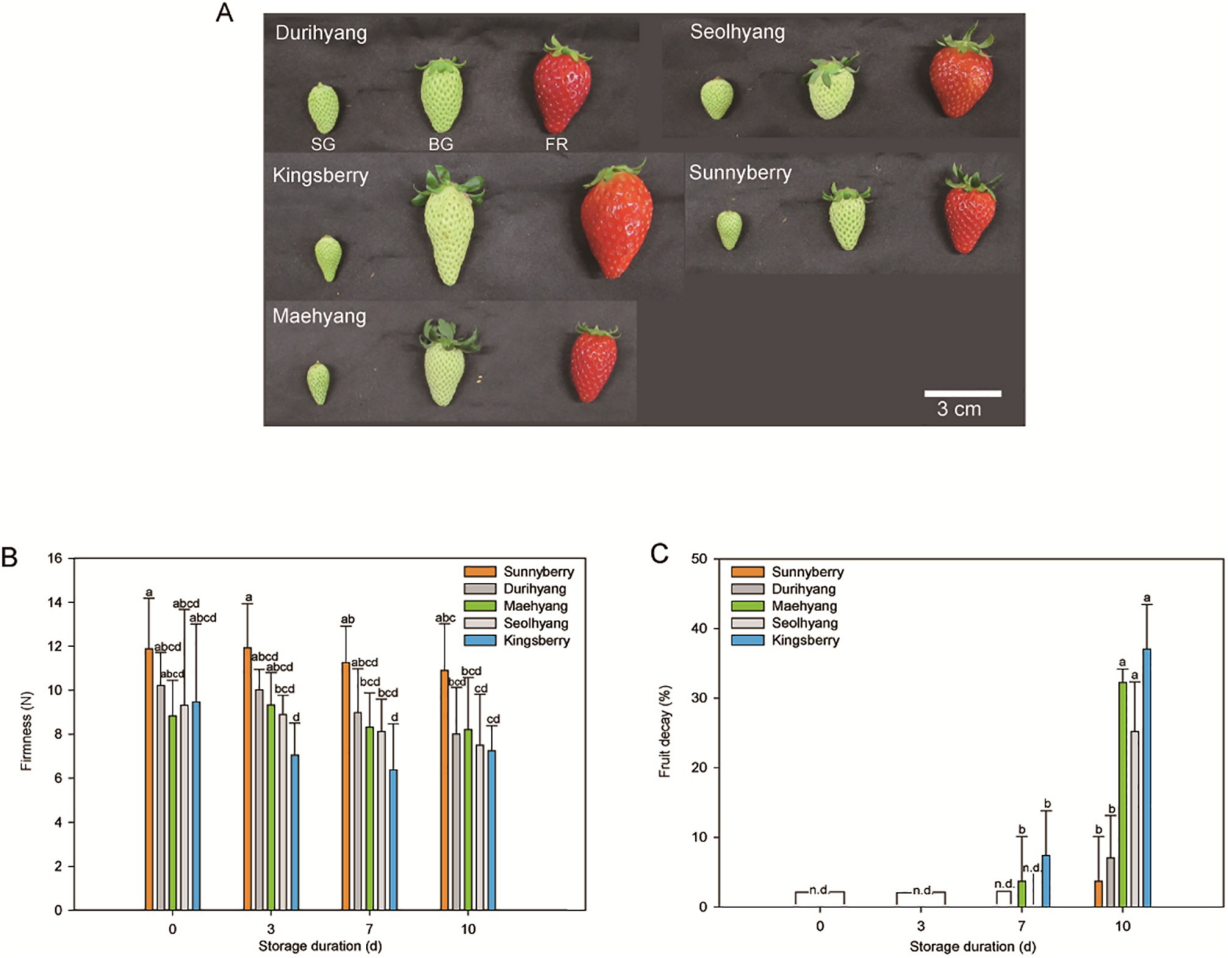
Postharvest fruit quality of five different strawberry cultivars during storage at 10°C for 10 d. (A) Appearances of the strawberry cultivars at the time of harvest. Samples at the small-green (SG), big-green (BG), and full-red (FR) stages were harvested, and fruit at the FR stage was used for storability evaluation. (B and C) Fruit firmness (N) and fruit decay rates (%) of the five cultivars during storage. n.d., non-detected. The data are expressed as the mean ± SD. Letters indicate significant differences at *P* < 0.05 in two-way ANOVA followed by the post-hoc Tukey’s HSD. Statistics can be found in S2 Table.

Fruit from five strawberry (*Fragaria* × *ananassa* Duch.) cultivars, namely ‘Durihyang’, ‘Kingsberry’, ‘Maehyang’, ‘Seolhyang’, and ‘Sunnyberry’, were cultivated in a glasshouse in the Strawberry Research Institute (Nonsan, South Korea) in 2018 and 2019. In mid-September, strawberry seedlings were transplanted into the soil of a plastic greenhouse with a density of 9,000 plants ha^−1^. Temperatures were maintained at 15−25°C during the day and over 10°C (autumn) or 6°C (winter) during the night. Fruits were harvested in the middle of March. Crossing combinations of the five Korean strawberry cultivars is presented in S1 Table. Fruit was uniformly selected according to size and coloration, corresponding to small-green (SG), big-green (BG), and fully-red (FR) fruit (Fig 1A). After immediately transporting the fruits to our laboratory, about ten fruits for each cultivar at each developmental stage were either directly measured for quality indicators or frozen in liquid nitrogen and stored at −80°C for subsequent transcriptome and metabolome analyses. Fruits harvested in 2018 were used for most of the experiments, except for fruit decay rate which was measured with fruits harvested during both years.

### Fruit firmness and decay rate measurements

Forty fruits from each cultivar at the FR stage were randomly picked and held in a storage chamber at 10°C for 10 d to compare postharvest fruit firmness (N) and decay rates (%). The relative humidity of the storage chamber was maintained at 85–90% during the 10-d incubation period. Ten strawberry fruits from each cultivar at the FR stage were randomly selected to measure firmness at 0, 3, 7, and 10 d after storage at 10°C using the CT-3 texture analyzer (Brookfield Co., Middleborough, MA, USA). To measure the fruit firmness, the equatorial plane of each strawberry fruit was analyzed using a flat probe (100 mm diameter) at a speed of 2 mm s^−1^ and a strain of 5 mm. Data from ten biological replicates were expressed as the mean ± standard deviation (SD).

Based on visual characteristics, the fruit was considered to be decayed if 10% of the fruit surface area showed damage from fungal infection or rot. The decay rate was calculated as a percentage by dividing the number of decayed fruits by the total number of fruits. The decay rate was calculated using a total of three biological replicates harvested between 2018 and 2019, and the data were expressed as the mean ± SD of these replicates.

### RNA extraction and cDNA synthesis

Frozen fruit was ground into a fine powder in liquid nitrogen. Total RNA was isolated from 0.1 g of powdered tissues using a Ribospin Seed/Fruit kit (GeneAll, Seoul, South Korea) according to the manufacturer’s instructions with DNase treatment (GeneAll) to eliminate possible DNA contamination. The purity of RNA was assessed both by agarose gel electrophoresis and the A260:A230 and A260:A280 ratios using a microplate spectrophotometer (BioTek Epoch, Winooski, VT, USA). Extracted total RNA was used for transcriptome analysis, and cDNA was synthesized for quantitative real-time PCR (qPCR) analysis. cDNA was synthesized using 500 ng of the total RNA and a ReverTra Ace qPCR RT Master Mix (Toyobo, Osaka, Japan) following the manufacturer’s instructions.

### Transcriptome and single-nucleotide polymorphism analysis by RNA-Seq

**Fig 2:**
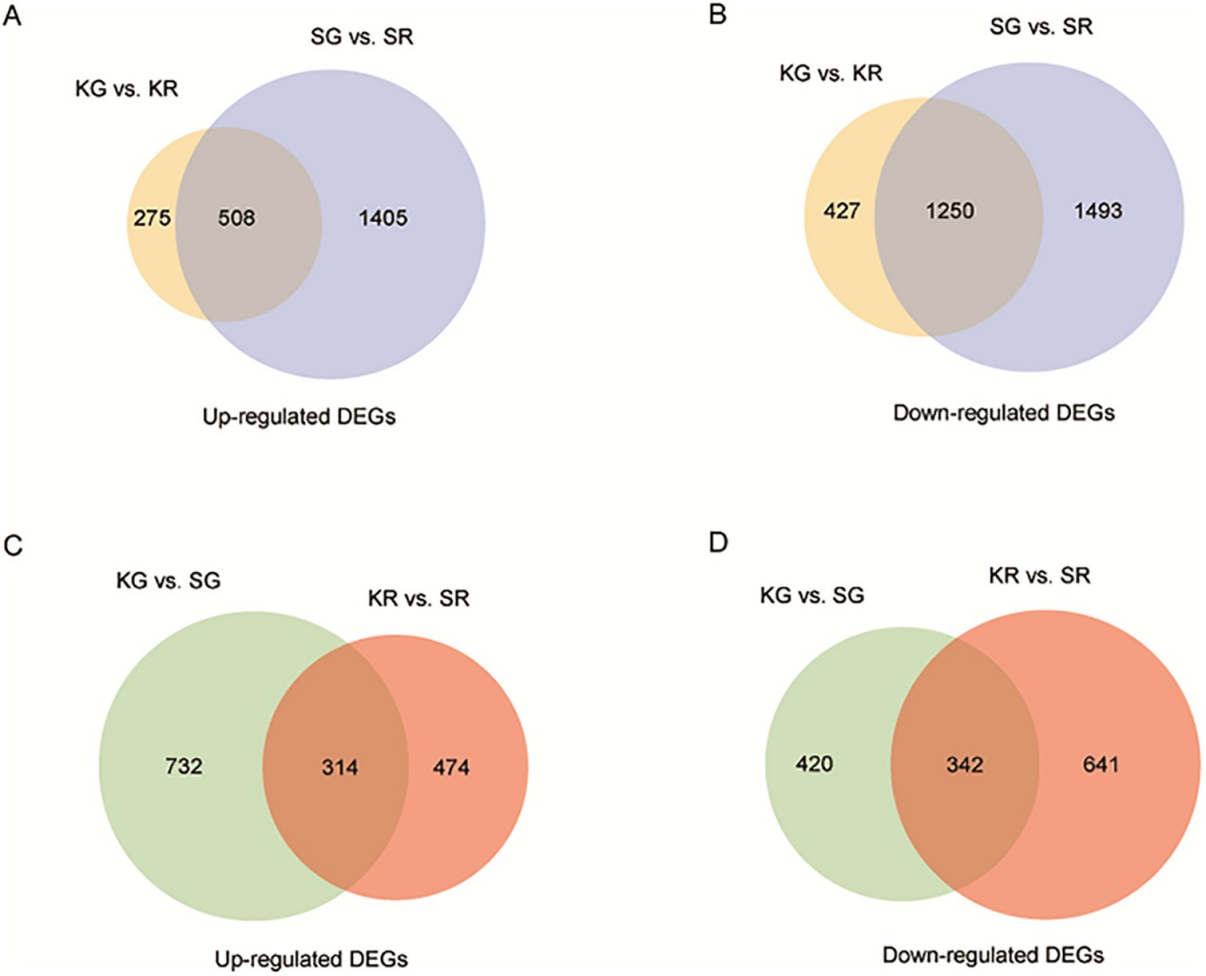
Transcriptomes of the ‘Kingsberry’ and ‘Sunnyberry’ cultivars. (A, C) Number of up-regulated differentially expressed genes (DEGs) between ‘Kingsberry’ and ‘Sunnyberry’ at the big-green (BG) and full-red (FR) stages. (B, D) Number of down-regulated DEGs between ‘Kingsberry’ and ‘Sunnyberry’ at the BG and FR stages. Abbreviations: KG, ‘Kingsberry’ at the BG stage; KR, ‘Kingsberry’ at the FR stage; SG, ‘Sunnyberry’ at the BG stage; SR, ‘Sunnyberry’ at the FR stage.

Total RNA from two cultivars (‘Kingsberry’ and ‘Sunnyberry’) at two developmental stages (BG and FR) was used for RNA-Seq with three biological replicates. RNA-Seq was performed on the HiSeq 2500 platform (Illumina, San Diego, CA, USA) using 151-bp paired-end reads at the National Instrumentation Centre for Environmental Management (NICEM), Seoul National University, South Korea. The raw data were deposited in the NCBI Short Read Archive (PRJNA564159). Low-quality (< Q20), adaptor, and barcode sequences were trimmed out by Trim Galore v.0.4.4 with the parameter ‘—gzip–paired’. The cleaned reads were mapped to the FAN_r1.1 reference genome [22] (S3 Table), and transcript levels were calculated as transcripts per million (TPM) using CLC Genomics Workbench v11 (Qiagen, Hilden, Germany) with default parameters (match score 1, mismatch cost 2, insertion cost 3, deletion cost 3, length fraction 0.5, and similarity fraction 0.8). Differentially expressed genes (DEGs) were obtained using two criteria, false-discovery rate *P* < 0.01, |fold-change| > 2, and a minimum expression of 0.3 TPM for four combinations; ‘KG vs. SG’, ‘KR vs. SR’, ‘KG vs. KR’, and ‘SG vs. SR’ (Fig 2). The transcripts were annotated according to Gene Ontology (GO) terms using Blast2GO 5.2 and the NCBI database [34]. Cleaned reads were also used for detecting single-nucleotide polymorphisms (SNPs). Variant calling was performed with CLC Genomics Workbench v11 using a sequencing depth > 10X.

### qPCR analysis

Synthesized cDNAs were diluted 1:10 with water, and 1 μL of each diluted cDNA was analyzed by qPCR. Mixtures (total volume of 10 μL) containing 0.5 μL of each primer (10 pM) and 2 μL of 2× Real-Time PCR Master Mix containing SYBR Green 1 (BioFACT, Daejeon, South Korea) were used. qPCR was performed with a CFX Connect Real-Time System (Bio-Rad, Hercules, CA, USA) under the following thermal cycling conditions: 95°C for 15 min followed by 40 cycles at 95°C for 20 s, 55°C for 40 s, and 72°C for 20 s. The primers (S4 Table) were designed using NCBI Primer-BLAST (https://www.ncbi.nlm.nih.gov/tools/primer-blast/). For each reaction, *C*_t_ values were normalized to that of the reference gene glyceraldehyde-3-phosphate dehydrogenase (AB363963.1) [35], and relative gene-expression levels were calculated using the 2^−ΔΔCt^ method [36]. The amplification efficiency for each gene was calculated using a standard curve method with following formula: E = (10^−1/slope^-1). The slope was derived from the 10-fold serial dilution of the cDNA samples. The analysis was performed with three biological replicates of randomly selected fruit for each cultivar at each time point. Three technical replicates of each sample were applied for checking the reproducibility.

### Untargeted polar-metabolite analysis

Fruit from ‘Kingsberry’ and ‘Sunnyberry’ at the BG and FR stages (three biological replicates each) was used for metabolite analysis. Untargeted-metabolite analysis was performed using gas chromatography-mass spectrometry (GC-MS). Polar metabolites were extracted using an established method [37], with specific modifications. Briefly, fine powder from frozen fruit (50 mg) was vortexed with 1.2 mL methanol and mixed with 20 μL of ribitol (2.0 g L^−1^ in water) as an internal quantitative standard. Then, each extract was dried in a vacuum concentrator for 1 h. For derivatization, 50 μL of methoxyamine hydrochloride (10.0 g L^−1^ in water) was added to each dried sample and incubated for 2 h at 37°C while shaking at 200 rotations/min. N-methyl-N-(trimethylsilyl) trifluoroacetamide (100 μL) was added to 40-μL sample aliquots and incubated for 30 min at 37°C. Then, 1 μL of each sample was analyzed using a GC-MS ISQ LT system with a Triplus100 liquid sampler (Thermo Fisher Scientific, Waltham, MA, USA).

Raw GC-MS data were analyzed with Xcalibur Qual Browser software version 4.2.47 (Thermo Fisher Scientific). Metabolites were annotated by comparing the mass spectra to the mass-spectrometry library from the NIST Mass Spectra Search Program version 2.0. Data processing was performed with Xcalibur Processing Setup version 4.2.47 (Thermo Fisher Scientific). Metabolite content was quantified by comparing the m/z peak areas to that of an internal standard and normalized to each sample weight. All metadata and information pertaining to metabolite profiling is provided in S5 Table. The identification level was reported according to Sumner et al. [38].

### Free amino acid analysis

Free amino acids were analyzed by HPLC as described previously [39], with some modifications. First, 100 mg of each frozen fruit powder was vortexed with 1.2 mL of 5% trichloroacetic acid and sonicated for 30 min at room temperature. After centrifugation at 15,000 × *g* for 15 min, 1 mL of each supernatant was filtered through a 0.45-μm polyvinylidene fluoride membrane filter (TaeshinBio, Seoul, South Korea). Then, 1 μL of each filtrate was mixed with 5 μL of borate buffer (Agilent Technologies, Palo Alto, CA, USA). For derivatization, 1 μL each of *o*-phthalaldehyde (OPA) and fluorenylmethyloxycarbonyl chloride (FMOC) reagents (Agilent Technologies) was added to each sample (in that order), and then the samples were mixed. After dilution with 32 μL of water, 0.5 μL of each sample was injected to a Dionex Ultimate 3000 HPLC instrument (Dionex, Sunnyvale, CA, USA) equipped with a UV detector and an Agilent 1260 infinity FL detector (Agilent Technologies). The emission and excitation wavelengths used with the FL detector were 450 nm and 340 nm for the OPA analysis, and 305 nm and 266 nm for the FMOC analysis, respectively. The wavelength used with the UV detector was 305 nm. HPLC was performed using an Inno C18 column (4.6 mm × 150 mm, 5-μm particle size, YoungJin Biochrom, Seongnam, South Korea) at 40°C. NaH_2_PO_4_ (40 mM, pH 7.0) and acetonitrile:methanol:H_2_O (45:45:10 v/v %) were used as mobile phases A and B, respectively. Different amino acids were used as external standards. The content of amino acids was calculated based on their peak areas and equivalents of standard compounds. They were then normalized to each sample’s fresh weight.

### Fatty acid analysis

Fatty acids were extracted from freeze-dried strawberry fruit and analyzed using GC following an established method [40], with some modifications. Pentadecanoic acid was added as an internal quantitative standard to 100 mg of freeze-dried fruit powder. They were mixed with 2 mL of methylation buffer containing methanol, benzene, dimethoxypropane, and sulfuric acid at a ratio of 39:20:5:2. After adding 1 mL of heptane, the mixture was incubated at 80°C for 2 h and then at room temperature for 1 h. Next, 1 μL of supernatant was injected into an Agilent 7890A GC instrument equipped with a DB-23 column (60 mm × 0.25 mm × 0.25 μm, Agilent Technologies) and a flame-ionization detector. The flame-ionization detector was operated at 280°C with a flow rate of 35 mL min^−1^ for H_2_, 350 mL min^−1^ for air, or 35 mL min^−1^ for He, and the injector was operated at 250°C. GC analysis was performed under the following temperature conditions: holding for 1 min at 50°C followed by heating to 130°C at 15°C min^−1^, heating to 170°C at 8°C min^−1^, heating to 215°C at 2°C min^−1^, and holding at 215°C for 10 min. The fatty acid contents were calculated by comparing their peak area to that of an internal standard. They were then normalized to each sample’s fresh weight.

### Metabolic pathway analysis

Metabolic pathways were illustrated with PathVisio 3.3.0 software using log_2_ (fold-change) values for DEGs and the metabolite contents of ‘Kingsberry’ and ‘Sunnyberry’ at the BG and FR stages. The metabolic pathways were drawn based on the Kyoto Encyclopedia of Genes and Genomes (KEGG) and pathway analysis with MetaboAnalyst 4.0 (https://www.metaboanalyst.ca/). Significant differences in metabolite content were tested with a two-sample t-test using SPSS version 25.0 (IBM, Armonk, NY, USA) and are reported at *P* < 0.05, *P* < 0.01, and *P* < 0.001.

### Statistical analysis

The relative mRNA-expression data are expressed as the mean ± SD of the three biological replicates. Two-way ANOVA was used to test the effect of storage days, cultivars, and storage days × cultivars interaction on fruit firmness with SPSS version 25.0. Metabolite-quantification data were analyzed using MetaboAnalyst version 4.0. After normalizing the data with auto-scaling, principal component analysis (PCA) and heatmap cluster analysis were performed.

## Results

### Postharvest fruit storability of the five strawberry cultivars

Five strawberry cultivars that are popular in South Korea were characterized in the present study, including ‘Seolhyang’ (the leading cultivar) and four newly developed cultivars. The cultivars showed slightly different fruit shapes, sizes, and colors at the FR stage (Fig 1A). ‘Kingsberry’ had a longer shape at the BG stage and the biggest fruit at the FR stage, compared to other cultivars. ‘Kingsberry’ had a lighter color at the FR stage, whereas ‘Durihyang’ had the darkest skin color.

The fruit firmness differed depending on the cultivar, however, it was not significant at harvest (0 d; Fig 1B). Among the cultivars, ‘Sunnyberry’ had the highest firmness (10.9 N) after 10 d of storage, and the firmness was maintained for the longest time during the storage period. In contrast, the firmness of ‘Kingsberry’ was 7.2 N after 10 d of storage, representing the lowest firmness among the five cultivars. ‘Seolhynag’ had the second lowest firmness with 7.5 N after 10 d of storage. The firmness was apparently specific to the cultivar rather than the storage duration throughout the storage period.

The fruit decay rate of ‘Sunnyberry’ was 3.7% over 10 d, the lowest among the five cultivars, while that of ‘Durihyang’ was 7% (Fig 1C). In contrast, the fruit of the other three cultivars rapidly decayed during 7–10 d of storage compared to ‘Sunnyberry’ and ‘Durihyang’. With ‘Seolhyang’, 25.2% of the fruit had decayed after 10 d, and 32.2% and 37.0% of the ‘Maehyang’ and ‘Kingsberry’ fruit decayed within 10 d, respectively. The ‘Kingsberry’ fruit was the first to decay, beginning after 5 d of storage. Therefore, ‘Sunnyberry’ and ‘Kingsberry’ were considered to have the best and worst fruit storability among the five cultivars, respectively, and these two cultivars were selected for further transcriptome and metabolite analysis to reveal postharvest storability-related TFs in strawberry fruit.

### Genetic distance between the ‘Kingsberry’ and ‘Sunnyberry’ cultivars

We obtained an average of 53 million reads per sample, ranging from 38 to 90 million reads among 12 samples (2 cultivars × 2 developmental stages × 3 biological replicates). The reads were mapped to the reference genome (http://strawberry-garden.kazusa.or.jp) with an average mapping rate of 63.6%, of which 89% were mapped to genic regions. The relatively low mapping rates showed genetic distances among the samples, and the reference cultivar genome lacked satisfactory quality due to its octoploid origin [22,41].

The genetic distances among the two cultivars and the reference cultivar were estimated through SNP comparisons. Out of 408,508 SNPs, 1.6-fold more SNPs were detected compared to the reference cultivar (316,536 and 308,619 SNPs for ‘Kingsberry’ and ‘Sunnyberry,’ respectively) than between the cultivars (192,052 SNPs) tested in the present study (S1 Fig). Based on the breeding history of the cultivars (S1 Table), such genetic distances are reasonable. The reference genome was built with the Japanese octoploid strawberry cultivar ‘Reikou’, and it partly contributed to the breeding of the Korean cultivars used in our study. For example, the maternal parent of ‘Kingsberry’ is ‘Akihime’, a Japanese cultivar, 25% of which originated from ‘Reikou’. Since the strawberry cultivars were vegetatively propagated, the breeding program employed many common popular cultivars, and their pedigree shared historically elite cultivars, such as ‘Reikou’.

### Overall transcriptome characteristics of the ‘Kingsberry’ and ‘Sunnyberry’ cultivars

The gene expression values among the three biological replicates were highly correlated. The average correlation coefficient (*r*^*2*^*)* values among the three biological replicates were 0.91 and 0.90 for the ‘Kingsberry’ and ‘Sunnyberry’ at the BG stage, respectively. At the FR stage, the *r*^*2*^ values increased to 0.98 and 0.97 in ‘Kingsberry’ and ‘Sunnyberry’, respectively. The average and standard errors of TPM values from the three biological replicates were used for further analysis.

The *r*^*2*^ value between ‘Kingsberry’ and ‘Sunnyberry’ was 0.85 at the FR stage but was only 0.61 at the BG stage. Both cultivars had more variation in their transcriptome profiles at the BG stage than at the FR stage. We found 1,484 ‘Sunnyberry’-specific and 2,114 ‘Kingsberry’-specific transcripts in the fruit tissues. Out of over 26,600 transcripts, the abundance of 29.4% (7,828) and 15.4% (4,164) of the transcripts changed during ripening in ‘Sunnyberry’ and ‘Kingsberry’, respectively (false-discovery rate *P* < 0.05). Such inconsistencies between the cultivars could explain the considerable differences in fruit storability.

Thus, we focused on the differences during ripening between the two cultivars. To minimize false positives, we used strict criteria to define DEGs for further analysis, namely false-discovery rate *P* < 0.01 and |fold-change| > 2. Using these criteria, 4,656 and 2,460 transcripts were defined as DEGs between the BG and FR stages for ‘Sunnyberry’ and ‘Kingsberry’, respectively (Fig 2A and 2B; S6 and S7 Tables). In ‘Kingsberry’, 783 transcripts were up-regulated in the FR stage compared to the BG stage, and 1,677 transcripts were down-regulated in the FR compared to BG stage; whereas 1,913 and 2,743 transcripts were up- and down-regulated, respectively, in the FR stage compared to the BG stage in ‘Sunnyberry’. Among them, 1,758 (508 up-regulated and 1,250 down-regulated) genes were commonly altered in both cultivars. ‘Sunnyberry’ contained two-fold more DEGs with 1,405 and 1,493 ‘Sunnyberry’-specific up-regulated and down-regulated DEGs, respectively. When ‘Kingsberry’ and ‘Sunnyberry’ fruit were compared at the same stage, 1,520 and 1,403 genes were up- and down-regulated, respectively (Fig 2C and 2D; S8 and S9 Tables).

### Transcriptome differences between the ‘Kingsberry’ and ‘Sunnyberry’ cultivars

RNA-Seq analysis revealed the transcript profiles of ‘Kingsberry’ and ‘Sunnyberry’ at the BG and FR developmental stages. Receptacles and achenes generally increased in size during the BG stage. During the FR stage, cell growth terminated in the receptacles, flavonoids accumulated, and the achenes were fully matured. DEGs found between fruit at these two stages reflect developmental changes, including anthocyanin accumulation, cell wall softening, and plant-hormone signaling.

DEGs of both cultivars commonly showed alterations of anthocyanin biosynthesis-related genes (S2 Fig). Genes encoding anthocyanin biosynthesis enzymes, including phenylalanine ammonia-lyases, chalcone synthases, flavanone 3-hydroxylases, dihydroflavonol 4-reductases, anthocyanin synthases, and UDP-glucoflavonoid 3-O-glucosyltransferases (*UFGTs*) were detected as up-regulated DEGs at the FR stage, Furthermore, the fold-changes of these up-regulated DEGs were much higher in ‘Sunnyberry’ than in ‘Kingsberry’, which resulted from the lower transcript levels at the BG stage and the higher transcript levels at the FR stage of ‘Sunnyberry’. These tendencies were commonly observed with most of the anthocyanin-biosynthetic genes and their orthologues, including phenylalanine ammonia-lyase, chalcone synthase, chalcone isomerase, flavanone 3-hydroxylase, dihydroflavonol 4-reductase, anthocyanin synthases, and *UFGT2* (S2 Fig).

The transcript levels of cell wall-degrading enzymes also showed distinct patterns between both cultivars (S3 Fig). At the FR stage, ‘Kingsberry’ expressed higher transcript levels of pectinesterases and pectate lyases compared to ‘Sunnyberry’; while ‘Sunnyberry’ expressed more polygalacturonases and expansins than ‘Kingsberry’. Furthermore, most of these genes were more highly expressed at the BG stage in ‘Kingsberry’ than in ‘Sunnyberry’, indicating more active cell wall degradation from an early developmental stage in ‘Kingsberry’ compared to ‘Sunnyberry’.

Strawberry fruit ripening is highly correlated with the regulation of hormones, such as gibberellin and ABA (S4 Fig). Gibberellin 20-oxidase was commonly detected as a down-regulated DEG during ripening, whereas gibberellin 2-β-dioxygenase was only observed as a ‘Sunnyberry’-specific up-regulated DEG. ABA is known to act as a key regulator that accelerates the ripening of non-climacteric fruits, such as strawberries [8,9]. Both cultivars showed differences in gene repertoires in terms of ABA biosynthesis, perception, and signaling. Three different ABA receptors, including pyrabactin resistance/pyrabactin resistance-like receptors, magnesium-chelatase H subunit (*CHLH*)/ABA receptor (*ABAR*), and *GTG*/*GCR*, play roles in ABA signaling. ABAR pyrabactin resistance 1 was commonly detected as a down-regulated DEG in both cultivars, whereas another ABAR (*PYL12*) was detected as a down-regulated DEG in ‘Kingsberry’, however was an up-regulated DEG in ‘Sunnyberry’. Three *CHLH*/*ABAR* orthologues were detected as down-regulated DEGs in ‘Sunnyberry’, whereas only one was down-regulated in ‘Kingsberry’. Four *GCR2* orthologues were expressed in both cultivars. However, these four orthologues were down-regulated only in ‘Sunnyberry’, even though a decreasing tendency was also observed in ‘Kingsberry’. A downstream gene for ABA insensitive 5 (*ABI5*) was only detected as a down-regulated DEG in ‘Sunnyberry’, revealing a difference in ABA-responsive gene-expression.

Additionally, genes encoding pyruvate decarboxylases, which carry out the first enzymatic step involved in ethanol fermentation, were detected as ‘Sunnyberry’-specific up-regulated DEGs (S5 Fig). The downstream alcohol dehydrogenase and aldehyde dehydrogenase genes were commonly down-regulated in both cultivars, where more orthologous and more significant down-regulation was detected in ‘Sunnyberry’.

Genes showing the highest expression levels at the BG and FR stages were seed storage-related proteins annotated as legumin- or cruciferin-like and vinorine synthases, respectively (S10 and S11 Tables). It is notable that genes encoding vinorine synthases were significantly up-regulated in ‘Sunnyberry’, but not in ‘Kingsberry’, showing differences in ripening physiology between both cultivars.

### Differences in fruit ripening between the ‘Kingsberry’ and ‘Sunnyberry’ cultivars revealed by GO analysis

To better understand the characteristics of DEGs and differences between both cultivars, we performed GO-enrichment analysis with the DEGs of both cultivars between the BG and FR stages. Both cultivars shared nine out of the top-10 GO terms for their down-regulated DEGs at the FR stage and showed consistent transcriptome changes in genes related to carbohydrate metabolism, photosynthesis, and organic acid biosynthesis (Table 1).

**Table 1:** Top 10 enriched GO terms in level 4 of ‘Sunnyberry’ and ‘Kingsberry’ DEGs between the big-green and full-red stages.

	GO ID	GO Name	FDR *P*	No. in DEGs	Expected No.
‘Sunny-berry’ UP	GO:1901576	organic substance biosynthetic process	1.84E-66	373	135.60
	GO:0022613	ribonucleoprotein complex biogenesis	2.78E-59	113	13.80
	GO:0044283	small molecule biosynthetic process	1.54E-45	119	21.69
	GO:0044249	cellular biosynthetic process	1.94E-45	316	128.70
	GO:1901564	organonitrogen compound metabolic process	1.64E-30	329	164.59
	GO:0006810	transport	4.92E-28	178	67.36
	GO:0006082	organic acid metabolic process	8.38E-20	144	58.50
	GO:0005975	carbohydrate metabolic process	6.27E-19	106	36.79
	GO:0044550	secondary metabolite biosynthetic process	5.59E-18	28	2.44
	GO:0055086	nucleobase-containing small molecule metabolic process	7.61E-15	52	12.56
‘Sunny-berry’ DOWN	GO:0005975	carbohydrate metabolic process	3.90E-101	270	50.97
	GO:0015979	photosynthesis	3.95E-71	81	3.68
	GO:1901564	organonitrogen compound metabolic process	4.38E-56	505	234.74
	GO:0044262	cellular carbohydrate metabolic process	8.56E-49	140	27.97
	GO:0006091	generation of precursor metabolites and energy	3.49E-42	108	19.00
	GO:1901576	organic substance biosynthetic process	3.18E-39	401	194.80
	GO:0006082	organic acid metabolic process	1.70E-37	227	83.19
	GO:0005996	monosaccharide metabolic process	3.82E-37	74	9.45
	GO:1901575	organic substance catabolic process	1.43E-32	168	55.26
	GO:0070887	cellular response to chemical stimulus	3.28E-31	96	20.53
‘Kings-berry’ UP	GO:0044036	cell wall macromolecule metabolic process	8.46E-20	23	1.04
	GO:0042546	cell wall biogenesis	6.90E-19	24	1.34
	GO:1901576	organic substance biosynthetic process	1.15E-12	121	56.09
	GO:0071555	cell wall organization	1.48E-12	25	3.02
	GO:0045229	external encapsulating structure organization	3.34E-12	25	3.15
	GO:0044550	secondary metabolite biosynthetic process	1.11E-11	16	1.03
	GO:0044262	cellular carbohydrate metabolic process	1.62E-10	36	8.27
	GO:0071669	plant-type cell wall organization or biogenesis	3.07E-10	15	1.10
	GO:0005975	carbohydrate metabolic process	5.04E-10	49	15.18
	GO:0043062	extracellular structure organization	6.98E-08	7	0.15
‘Kings-berry’ DOWN	GO:0005975	carbohydrate metabolic process	3.79E-97	210	31.47
	GO:0015979	photosynthesis	7.36E-55	59	2.40
	GO:0044262	cellular carbohydrate metabolic process	1.60E-49	112	17.24
	GO:0005996	monosaccharide metabolic process	2.83E-36	60	5.86
	GO:0006091	generation of precursor metabolites and energy	4.81E-34	77	11.80
	GO:1901564	organonitrogen compound metabolic process	3.20E-23	278	144.60
	GO:1901576	organic substance biosynthetic process	1.16E-20	236	119.82
	GO:0006810	transport	1.25E-20	147	59.25
	GO:0006082	organic acid metabolic process	7.82E-19	130	51.36
	GO:0070887	cellular response to chemical stimulus	2.21E-16	55	12.80

Down-regulated genes related to photosynthesis included chlorophyll and photosystem-related proteins, whose expression levels were almost eliminated at the FR stage, showing chlorophyll degradation during ripening as previously reported [42]. For the up-regulated DEGs, two of the top-10 GO terms were shared between both cultivars (Table 1). The up-regulated DEGs of ‘Kingsberry’ included three cell wall-related terms (cell wall macromolecule metabolic process, cell wall biogenesis, and cell wall organization) that were not up-regulated in ‘Sunnyberry’. Instead, the GO terms ribonucleoprotein complex biogenesis, small molecule biosynthetic process, cellular biosynthetic process, and secondary metabolite biosynthetic process were associated with the up-regulated DEGs in ‘Sunnyberry’.

### Metabolite changes in the ‘Kingsberry’ and ‘Sunnyberry’ during fruit ripening

**Fig 3:**
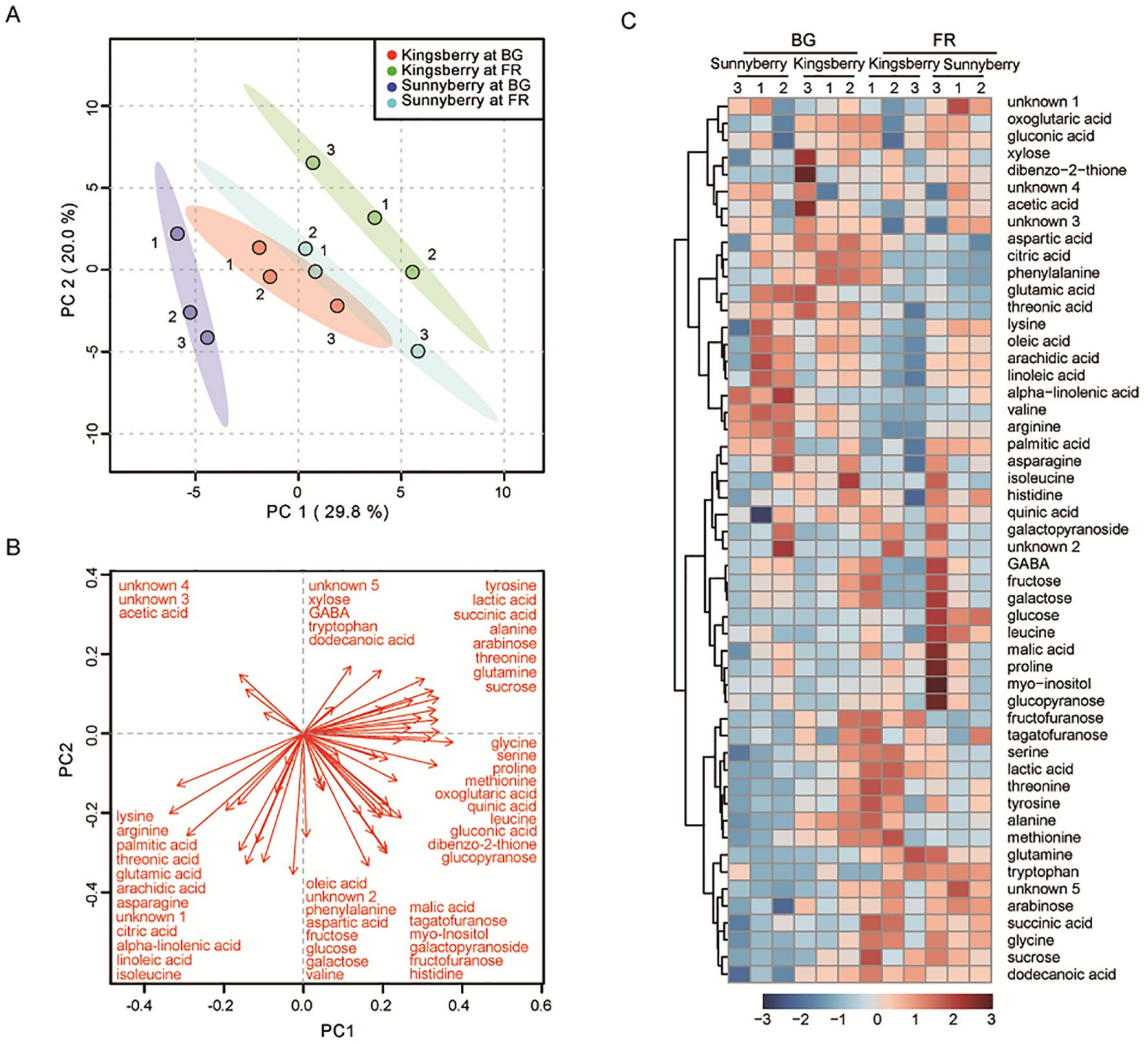
Metabolic differences between the ‘Kingsberry’ and ‘Sunnyberry’ cultivars at the big-green (BG) and full-red (FR) fruit-development stages. (A) PCA score plot. (B) PCA loading plot. (C) Heatmap of 46 metabolites identified and normalized with auto-scaling. PCA, principal component analysis.

We also performed fruit-metabolite analysis with both cultivars to reveal their different ripening patterns. Forty-six metabolites were identified and quantified by targeted and untargeted analysis. To verify the metabolic characteristics of the samples, PCA was conducted based on the metabolite content. The first and second principal components accounted for 29.8% and 20% of the variance in the dataset, respectively (Fig 3A). The samples were well separated by the combination of PC1 and PC2. Fruit at the FR stage of both cultivars was represented on the upper right side of the plot, which was well separated from the BG stage samples. The ‘Kingsberry’ samples were placed on the upper right side of the ‘Sunnyberry’ samples for both stages, suggesting that the cultivar-specific metabolite profiles were consistent at both the BG and FR stages. In PC1 the largest positive associations were observed with serine, glycine, and sucrose; while the largest negative associations were with lysine, arginine, and palmitic acid (Fig 3B).

Heatmap analysis showed metabolic changes based on the relative metabolite concentrations (Fig 3C), and a comparison of metabolite accumulation between BG and FR stages for each cultivar is shown in S12 Table. The metabolic profiles of sugars and organic acids differed for each cultivar. The content of saccharides, including galactose, fructose, and glucose, did not significantly change during ripening. However, the sucrose content clearly increased during ripening, and a higher sucrose content was detected in ‘Kingsberry’ than in ‘Sunnyberry’, especially at the BG stage (Fig 3C; S12 and S13 Tables). Additionally, ‘Sunnyberry’ showed significantly increased xylose and arabinose content during ripening but ‘Kingsberry’ did not. The citric acid content was higher in ‘Kingsberry’ at both stages, whereas the malic acid content was higher at the FR stage of ‘Sunnyberry’ compared to ‘Kingsberry’. The succinic acid content was higher in ‘Kingsberry’ than in ‘Sunnyberry’ at the BG stage, whereas that of ‘Sunnyberry’ increased significantly during ripening.

The other prominent changes that occurred during ripening were in the amino acid levels. The contents of serine, glycine, threonine, tryptophan, tyrosine, and alanine increased during ripening in both cultivars, but the overall content of these ripening-related amino acids was much higher in ‘Kingsberry’ than in ‘Sunnyberry’. However, the lysine and arginine contents were higher in ‘Sunnyberry’ than in ‘Kingsberry’ and commonly decreased in both cultivars during ripening. ‘Kingsberry’ also showed higher histidine, aspartic acid, and phenylalanine contents, especially at the BG stage. Additionally, more asparagine, arginine, and lysine contents were detected in ‘Sunnyberry’ at both stages, whereas leucine content increased rapidly in ‘Sunnyberry’ at the FR stage.

Fatty acid composition also changed during fruit ripening. Most fatty acids including palmitic acid, arachidic acid, oleic acid, linoleic acid, and alpha-linolenic acid decreased in both cultivars at the FR stage. The abundance of dodecanoic acid slightly increased during fruit ripening. The contents of fatty acids (except for dodecanoic acid) were higher in ‘Sunnyberry’ compared to ‘Kingsberry’ at both stages. These metabolic changes, including sugars, organic acids, amino acids, and fatty acids revealed common but cultivar-specific ripening processes between both cultivars.

### Combining transcriptomic and metabolomic analysis reveals changes during strawberry ripening

**Fig 4:**
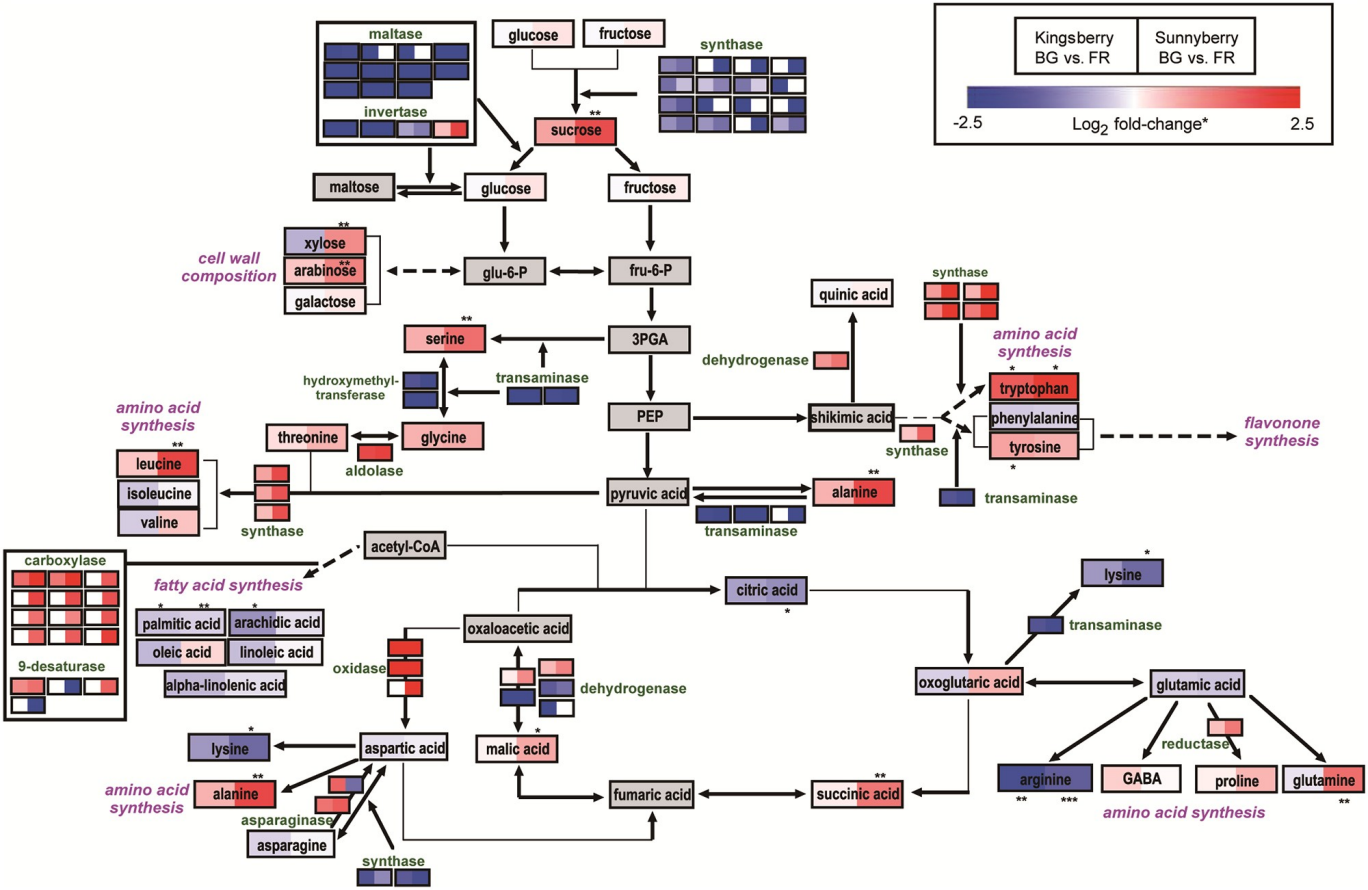
Pathway analysis based on transcriptome and metabolome data for the ‘Kingsberry’ and ‘Sunnyberry’ cultivars at the big-green (BG) and full-red (FR) stages. Gene expression or metabolite content was expressed as log_2_ fold-change between BG and FR stages for each cultivar. Significant differences in metabolite content were reported as *, **, and *** at *P* < 0.05, *P* < 0.01, and *P* < 0.001, respectively.

Identified metabolites including sugars, sugar acids, and amino acids were mapped to the sugar conversion, glycolysis, TCA cycle, amino acid metabolism, and lipid metabolism pathways. Enzymatic genes exhibiting differential expression in the KEGG annotation were also included in the pathway (Fig 4). This combined analysis provided an overview of the biological changes occurring during strawberry fruit ripening, which can be summarized as follows: (1) incremental increase in sugars, particularly in sucrose, was observed during ripening. ‘Sunnyberry’ showed higher fold-change in sugar content, which corresponded with invertase expression; (2) the content of several amino acids, such as tryptophan, alanine, leucine, etc. was observed to be accumulated with up-regulation of amino acid synthase; and (3) substrates for cell wall components, such as xylose and arabinose, exhibited differential accumulation during ripening and between the two cultivars.

### Possible TFs related to the postharvest storability of strawberry fruit

We further focused on genes that can be used as indicators of fruit storability. Candidate genes were selected based on the following criteria: (1) DEGs between the BG and FR stages, (2) DEGs between both cultivars at the same developmental stages, and (3) genes that can regulate multiple downstream genes, such as TFs. Based on the first two criteria, 1,798 genes were selected. Among them, many senescence-associated genes, ABA, and ethylene-related genes were included, showing that such criteria can enrich for senescence-related genes, which can be related to fruit storability. The third category narrowed the candidates to 85 genes (Table 2). The candidate genes included eleven *WRKY*s, two *NAC*s, three ABA-related genes, and six ethylene-related TF genes, and several of these genes were selected for validation by qPCR analysis.

**Table 2:** Candidate transcription factors for storability prediction from transcriptome comparison of the two cultivars, ‘Sunnyberry’ and ‘Kingsberry’.

Category	Annotations	Transcript IDs
AP2	AP2/ERF, RAV1-like, AIL5	FAN_iscf00054961.1.g00001.1, FAN_iscf00193262.1.g00001.1, FAN_iscf00134037.1.g00003.1
B3	ABI3, FUS3	FAN_iscf00351130.1.g00001.1, FAN_iscf00018985.1.g00001.1, FAN_iscf00128725.1.g00001.1
bHLH	bHLH149, bHLH155, bHLH62, UNE10	FAN_iscf00165139.1.g00001.1, FAN_iscf00053249.1.g00001.1, FAN_iscf00064718.1.g00004.1, FAN_iscf00191154.1.g00002.1, FAN_iscf00207814.1.g00001.1, FAN_iscf00298710.1.g00001.1
bZIP	ABI5, bZIP11, OHP2, MIP1	FAN_iscf00044476.1.g00001.1, FAN_iscf00008317.1.g00001.1, FAN_iscf00177814.1.g00001.1, FAN_iscf00021766.1.g00001.1, FAN_iscf00025369.1.g00001.1
ERF	ERF003-like, ERF105-like, ERF106-like, RAP2-4-like	FAN_iscf00295177.1.g00001.1, FAN_iscf00371725.1.g00001.1, FAN_iscf00004671.1.g00001.1, FAN_iscf00376638.1.g00001.1, FAN_icon20045513.1.g00001.1, FAN_iscf00307570.1.g00001.1
Homeo-domain	BEL1-like2, Knotted1-like LET6, SBH1, ATHB-13-like, ATHB-6-like, HAT4-like	FAN_iscf00036070.1.g00001.1, FAN_iscf00161053.1.g00001.1, FAN_iscf00178770.1.g00001.1, FAN_iscf00017690.1.g00001.1, FAN_iscf00274596.1.g00001.1, FAN_iscf00035808.1.g00001.1
ID	ID2-like, ID5-like	FAN_iscf00087103.1.g00003.1, FAN_iscf00231262.1.g00002.1, FAN_iscf00005204.1.g00001.1
MADS	AGL29	FAN_iscf00381635.1.g00001.1
MYB	MYB6-like	FAN_iscf00125059.1.g00001.1
NAC	NAC83, NAC92-like	FAN_iscf00089695.1.g00001.1, FAN_iscf00019465.1.g00002.1,
WRKY	WRKY24, WRKY31, WRKY40, WRKY48, WRKY70	FAN_iscf00153105.1.g00001.1, FAN_iscf00094603.1.g00001.1, FAN_iscf00121985.1.g00001.1, FAN_iscf00149471.1.g00001.1, FAN_iscf00182457.1.g00001.1, FAN_iscf00243170.1.g00001.1, FAN_iscf00042597.1.g00001.1, FAN_iscf00264237.1.g00001.1, FAN_iscf00018791.1.g00001.1, FAN_iscf00020052.1.g00002.1, FAN_iscf00010757.1.g00002.1
ZFs	ATL21B, ATL2, ATL20, ATL79, ATL8, CCCH domain-containing protein, MYM-type protein 1-like, CONSTANS-LIKE 10-like, CONSTANS-LIKE 16, ZAT10-like, ZAT12-like	FAN_iscf00047361.1.g00001.1, FAN_iscf00116849.1.g00001.1, FAN_iscf00004491.1.g00001.1, FAN_iscf00297418.1.g00001.1, FAN_iscf00015150.1.g00001.1, FAN_iscf00198464.1.g00001.1, FAN_iscf00268627.1.g00001.1, FAN_iscf00030345.1.g00001.1, FAN_iscf00128969.1.g00001.1, FAN_iscf00133774.1.g00001.1, FAN_icon20045425.1.g00001.1, FAN_iscf00102253.1.g00001.1, FAN_iscf00091343.1.g00001.1, FAN_iscf00124002.1.g00001.1, FAN_iscf00126559.1.g00001.1, FAN_icon19832991.1.g00001.1, FAN_iscf00283340.1.g00001.1, FAN_icon20670808.1.g00001.1, FAN_icon19584887.1.g00001.1, FAN_iscf00053721.1.g00001.1, FAN_iscf00187446.1.g00001.1, FAN_iscf00027849.1.g00001.1, FAN_iscf00370698.1.g00001.1, FAN_iscf00384820.1.g00001.1, FAN_iscf00095610.1.g00001.1, FAN_iscf00159605.1.g00001.1, FAN_iscf00198102.1.g00002.1, FAN_iscf00318859.1.g00001.1, FAN_iscf00187920.1.g00001.1, FAN_iscf00228019.1.g00001.1, FAN_iscf00164977.1.g00002.1, FAN_iscf00341252.1.g00001.1
Etc.	NF-Y subunit A-4-like, NF-Y subunit B-3-like, WD40-like, HEC2-like	FAN_iscf00019146.1.g00002.1, FAN_iscf00168168.1.g00002.1, FAN_iscf00323551.1.g00001.1, FAN_iscf00134093.1.g00001.1, FAN_iscf00069284.1.g00002.1, FAN_iscf00078116.1.g00001.1,

Correlations between the transcriptome profiles and qPCR analysis were confirmed based on the expression levels of 13 genes (S6 Fig). Pearson’s correlation coefficient (*r*) was calculated as 0.947, indicating the coincidence of expression changes between both types of analysis.

**Fig 5:**
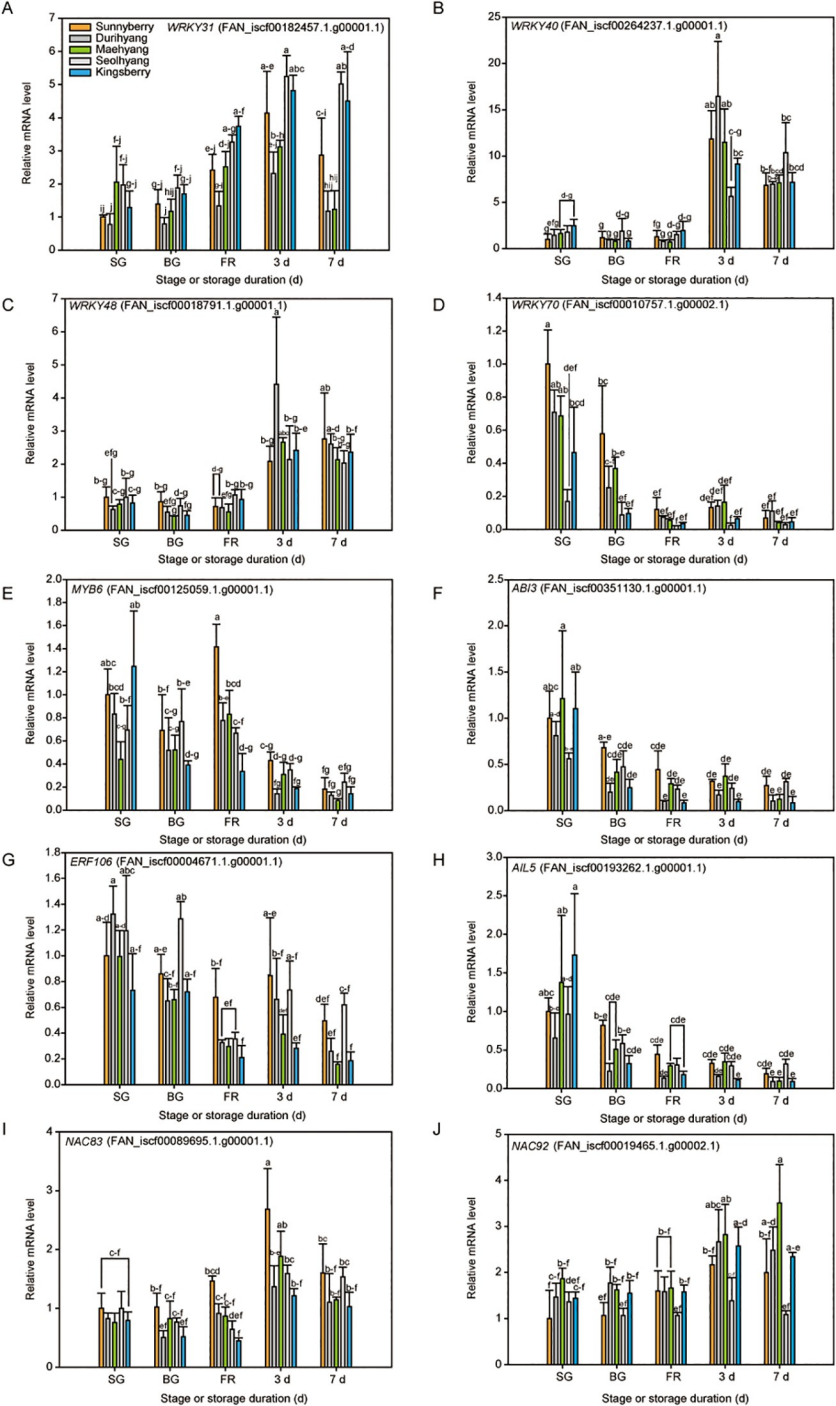
qPCR analysis of ten selected candidate genes related to the postharvest storability of strawberry fruit. The expression levels of (A) *WRKY31*, (B) *WRKY40*, (C) *WRKY48*, (D) *WRKY70*, (E) *MYB6*, (F) *ABI3*, (G) *ERF106*, (H) *AIL5*, (I) *NAC83*, and (J) *NAC92* in five different strawberry cultivars at the small-green (SG), big-green (BG), and full-red (FR) fruit-development stages and during storage at 10°C for up to 7 d are shown. The data are expressed as the mean ± SD of three biological replicates. Letters indicate significant differences at *P* < 0.05 in two-way ANOVA followed by the post-hoc Tukey’s HSD. Statistics can be found in S14 Table.

We tested whether the candidates were useful for predicting the postharvest fruit storability of five cultivars during the ripening and storage periods by qPCR analysis, and two distinct expression patterns were observed. *WRKY31*, *WRKY40*, *WRKY48*, *NAC83*, and *NAC92* were up-regulated during the ripening and storage periods, whereas *WRKY70*, *MYB6*, *ABI3*, *ETHYLENE-RESPONSIVE ELEMENT BINDING FACTOR106* (*ERF106*), and *AINTEGUMENTA-LIKE5* (*AIL5*) were down-regulated. Among them, ‘Seolhyang’ and ‘Kingsberry’, which have a short postharvest life, expressed more *WRKY31* mRNA than other cultivars, especially from the BG stage (Fig 5A). The expression of *WRKY40* was not significantly different among the cultivars at the early stage (Fig 5B). However, in the cultivars with a long postharvest life (‘Sunnyberry’ and ‘Durihyang’), *WRKY40* expression markedly increased at 3 d of storage compared to the cultivars with a short postharvest life (‘Seolhyang’ and ‘Kingsberry’), but the expression patterns were not different after 7 d of storage, except for ‘Seolhyang’. *WRKY48* expression also increased during the storage period, but only ‘Durihyang’ showed significantly increased *WRKY48* expression after 3 d of storage (Fig 5C). *WRKY70* expression was higher in the cultivars with a long postharvest life, such as ‘Sunnyberry’ and ‘Durihyang’ at early stages, when compared with ‘Seolhyang’ and ‘Kingsberry’ (Fig 5D).

‘Seolhyang’ and ‘Kingsberry’ showed continuously decreased *MYB6* expression during the ripening and storage periods, and the decreasing tendency was the strongest in ‘Kingsberry’ (Fig 5E). However, the other three cultivars showed increased *MYB6* expression during ripening, and the increasing tendency at the FR stage was the strongest in ‘Sunnyberry’, suggesting that increased *MYB6* expression during ripening affects the fruit storability. *NAC83* expression did not differ among the cultivars during the early stages, however, it was differentially expressed at the FR stage (Fig 5I). The higher the fruit storability, the higher the *NAC83*-expression level at the FR stage and at 3 d of storage. *ABI3*, *ERF106*, *AIL5*, and *NAC92* did not show differential expression patterns according to the postharvest storability among the five cultivars tested (Fig 5F–5H and 5J).

Among the selected possible indicators, *NAC83* is a homolog of *VND-INTERACTING2* (*VNI2*), which was identified in *A*. *thaliana* and plays an important role in regulating leaf senescence [43]. *VNI2* directly interacts with many *COLD-REGULATED* (*COR*) and *RESPONSIVE TO DEHYDRATION* (*RD*) genes, such as *COR15* and *RD29* in *Arabidopsis* and many other NAC TFs [43,44]. Thus, we tested whether the signaling mediated by *NAC83* is responsible for delaying fruit senescence by investigating the expression levels of *COR47* and *NAC2* (*RD26*).

*COR47* did not correlate well with *NAC83* expression (S7A Fig). *NAC2* was not differentially expressed at the SG stage among the five cultivars, but its expression increased coincidentally with that of *NAC83* during the storage period (S7B Fig). Additionally, ‘Sunnyberry’ maintained higher expression levels of *NAC2* and *NAC83* than the other cultivars, whereas ‘Kingsberry’ showed the lowest expression of these genes.

## Discussion

Transcriptome and metabolome changes during ripening were investigated in cultivars showing the highest (‘Sunnyberry’) and lowest (‘Kingsberry’) postharvest fruit storability (Fig 1B and 1C). Ripening and senescence are continuous processes involving complex developmental and physiological changes. Delaying senescence without inhibiting ripening is an ideal strategy for improving fruit storability without losing fruit quality. However, since both ripening and senescence continuously interact, selective control of one process should be based on a solid biological understanding.

Ripening is associated with various physiological characteristics, including sugar accumulation, cell wall softening, and anthocyanin accumulation. Such characteristics were evaluated in terms of transcriptome and metabolites changes by performing comparisons at two distinct developmental stages (BG and FR) in the present study. Strawberry fruit ripening was investigated from the early-receptacle stage to the fully mature stage, and the BG and FR stages effectively represent strawberry ripening [45]. The transcriptomes of ‘Kingsberry’ and ‘Sunnyberry’ showed conserved ripening-associated processes, including anthocyanin biosynthesis, cell wall metabolism, and hormonal regulation. Additionally, both cultivars showed clear differences that were possibly related to differences in postharvest fruit storability (S2–S5 Figs). Approximately 5−8% of the genes were thought to be cultivar-specific, and different orthologues were expressed during most ripening-related processes, showing significant genetic diversities between both cultivars.

The GO term ‘photosynthesis’ was commonly associated with down-regulated DEGs with both cultivars (Table 1), and genes corresponding to the ‘photosynthesis’ term included structural genes of PSI and PSII and chlorophyll-binding genes. Additionally, these genes were clearly diminished at the FR stage, indicating that chlorophyll degradation occurred during ripening. Chlorophyll degradation is a sign of senescence, indicating that ripening and senescence occur coincidentally during the transition from the BG to FR stages [46]. Hu et al. also observed similar changes in their transcriptome analysis of the cultivar ‘Toyonoka’ [24], for which they proposed that ubiquitin-mediated proteolysis plays an important role in strawberry ripening. We have identified DEGs related to ubiquitination in our analysis, however, further investigation is required to elucidate the associated relationship.

Differences in the ripening processes of both cultivars were also found in terms of metabolic changes. Increased sugar content is a general characteristic of fruit ripening [47], and the major sugars of strawberry fruit were reported to be glucose, fructose, and sucrose [48]. Among them, sucrose was the most increased saccharide in both cultivars during ripening, even though the expression levels of sucrose synthases decreased (Fig 4).

Sucrose accumulation during ripening was caused by down-regulation of various maltases and invertases that convert disaccharides to monosaccharides. Sucrose plays a role in regulating strawberry fruit ripening by transducing cross-signaling with ABA and stimulating phytohormone-induced ripening of non-climacteric fruit [8,9]. Additionally, down-regulation of the sucrose-synthase gene, *FaSS1*, delayed strawberry fruit ripening by lowering sucrose content, increasing fruit firmness, decreasing anthocyanin content, and down-regulating ripening-related genes [49]. In the present study, the lower sucrose content of ‘Sunnyberry’ at the BG stage (Fig 3C; S12 and S13 Tables) might have delayed ripening and postharvest senescence, when compared to ‘Kingsberry’.

Neutral sugars such as xylose, arabinose, and galactose are the main components of fruit cell walls. Thus, the loss of these sugars as well as pectins during ripening is related to cell wall loosening as well as fruit softening [5]. ‘Sunnyberry’, with the highest fruit firmness, showed significantly higher xylose and arabinose contents than ‘Kingsberry’, which had the lowest firmness at the FR stage (Fig 1B; Fig 4), suggesting that ‘Sunnyberry’ maintains firmness during ripening and senescence by accumulating neutral sugars in cell walls and by undergoing less cell wall degradation.

Fruit acidity usually decreases during ripening, mainly due to decreased citric acid, malic acid, and quinic acid contents [47]. Here, the citric acid, malic acid, and succinic acid contents differed for each cultivar during ripening (Fig 3C; S12 and S13 Tables), and these differences were related to DEGs encoding diverse enzymes. Genes encoding hydratases and dehydrogenases in the citrate cycle were differentially expressed between both cultivars and correlated with higher malic acid and succinic acid contents in ‘Sunnyberry’, especially at the FR stage (Fig 4). These findings indicated that ‘Sunnyberry’ maintains much higher acid levels than ‘Kingsberry’ during ripening, which could increase fruit storability by delaying ripening.

Diverse amino acids, such as asparagine, glutamine, arginine, glutamic acid, alanine, and aspartic acid, accumulate during fruit maturation [50]. Among them, asparagine, glutamine, and alanine mainly accumulate during fruit ripening [51]. ‘Kingsberry’ produced more ripening-related amino acids, including alanine and glutamine, whereas ‘Sunnyberry’ had a higher asparagine content (Fig 3C; S12 and S13 Tables). Both cultivars shared some common changes in amino acid composition during fruit ripening, but the profiles and contents differed for each cultivar, and these differences were highly correlated with the DEGs. The expression levels of enzymes that synthesize amino acids from glycolysis or citrate cycle products (transaminases, hydroxymethyltransferases, and amino acid synthases) changed, which could account for changes in the amino acid profiles of both cultivars (Fig 4).

Most fatty acids decreased during fruit ripening, but ‘Kingsberry’ showed a much more rapid decrease in fatty acid levels (Fig 3C; S12 and S13 Tables), which was related to DEGs encoding carboxylases and 9-desaturases involved in fatty acid biosynthesis (Fig 4). Since ‘Sunnyberry’ expressed these enzymes much more than ‘Kingsberry’, ‘Sunnyberry’ could maintain higher fatty acid levels during ripening.

We investigated key TFs that can cause such differences during fruit ripening and senescence. Among the tested candidates, *WRKY31*, *WRKY70*, and *NAC83* expression correlated with fruit storability (Fig 5A, 5D and 5I), suggesting their potential use as indicators of fruit storability.

Four WRKY TFs (*WRKY31*, *WRKY40*, *WRKY48*, and *WRKY70*) were differentially expressed among the five cultivars studied (Fig 5A–5D). Many WRKY TFs are induced by abiotic and biotic stresses [52]. For example, *WRKY18*, *WRKY40*, and *WRKY60* interact with one another and play different roles in defense responses to bacterial (*Pseudomonas syringae*) or fungal (*Botrytis cinerea*) infections [53]. *WRKY48* also participates in plant defense responses by lowering resistance to bacterial infection by *P*. *syringae* in *Arabidopsis* [54]. Here, five strawberry cultivars showed different fruit decay rates during storage (Fig 1C), and fruit decay mainly resulted from fungal infection. Although the roles of WRKY TFs in defense responses induced by fungal pathogens were not clearly revealed, their complex signaling mechanisms and expression profiles might directly or indirectly affect fruit decay.

*NAC83*, a *VNI2* homolog, is a TF of plant senescence. It integrates ABA signaling mediated by *ABI5* and *NAC92* and ethylene signaling through *EIN3* and *NAC29* to control the *Arabidopsis* leaf senescence [55]. Additionally, *VNI2* can negatively regulate *Arabidopsis* leaf aging by inducing downstream stress-responsive genes, such as *COR*s and *RD*s [43]. Here, ‘Sunnyberry’ showed higher expression of *NAC83* and its downstream target gene, *NAC2*, during ripening and senescence, whereas these genes showed the lowest expression in ‘Kingsberry’ (Fig 5I; S7B Fig). Thus, *NAC83* may regulate the delay of strawberry fruit senescence by interacting with *NAC2*. The expression levels of *NAC83* and *NAC2* suggest that such integration is conserved in strawberry fruit senescence and that the interactive signaling pathway can be exploited to manipulate strawberry fruit storability.

## Conclusions

We hypothesized that variations in strawberry cultivar storability is caused by differences in associated TFs. Here, we confirmed this hypothesis by showing that strawberry fruit ripening and senescence varied in terms of the transcriptome and metabolome, depending on the cultivar, suggesting that fruit storability can be improved by breeding and genetic modification. TFs, including *WRKY31*, *WRKY70*, and *NAC83* involved in strawberry fruit ripening were also highly related to senescence. A deeper understanding of ripening and senescence should enable a genetic basis for improving the postharvest storability of strawberry fruit, without delaying ripening or reducing fruit quality.

## Supporting information

S1 Fig Phylogenetic relationships between both cultivars and the reference cultivar ‘Reikou’ (strawberry-garden.kazusa.or.jp). (DOCX)

S2 Fig Transcript levels of differentially expressed genes in the anthocyanin biosynthesis pathway. (DOCX)

S3 Fig Transcript levels of differentially expressed genes related to cell wall softening. (DOCX)

S4 Fig Transcript levels of differentially expressed genes related to hormones. (DOCX)

S5 Fig Transcript levels of differentially expressed genes involved in ethanol fermentation. (DOCX)

S6 Fig Correlation between RNA-Seq and qPCR data, based on log2 (fold-change) differences between the ‘Kingsberry’ (red dots) and ‘Sunnyberry’ (black dots) cultivars at the big-green and fully-red stages. (DOCX)

S7 Fig Relative-expression levels of (A) *COR47* and (B) *NAC2* in five different strawberry cultivars at the small-green (SG), big-green (BG), and full-red (FR) developmental stages, or during storage at 10°C for up to 7 d. (DOCX)

S1 Table Crossing combination of five strawberry cultivars. (DOCX)

S2 Table Statistics for analysis of variance for effect of cultivar, storage duration, and their interaction on fruit firmness. (DOCX)

S3 Table Summary of RNA-Seq mapping. (DOCX)

S4 Table Sequences of primers used for qPCR analysis. (DOCX)

S5 Table Information for the polar metabolites annotated in the strawberry fruit using GC-MS. (DOCX)

S6 Table Gene expression profiles and differential gene expression analysis of ‘Kingsberry’ at the big-green and fully-red stages. (XLSX)

S7 Table Gene expression profiles and differential gene expression analysis of ‘Sunnyberry’ at the big-green and fully-red stages. (XLSX)

S8 Table Gene expression profiles and differential gene expression analysis of ‘Kingsberry’ and ‘Sunnyberry’ at the big-green stage. (XLSX)

S9 Table Gene expression profiles and differential gene expression analysis of ‘Kingsberry’ and ‘Sunnyberry’ at the fully-red stage. (XLSX)

S10 Table Most highly expressed transcripts (TPM > 1000 in one of the cultivars) in ‘Kingsberry’ and ‘Sunnyberry’ during the big-green (BG) stage. (DOCX)

S11 Table Most highly expressed transcripts (TPM >1000 in one of the cultivars) in ‘Kingsberry’ and ‘Sunnyberry’ in the fully-red (FR) stage. (DOCX)

S12 Table Metabolite accumulation at big-green (BG) and fully-red (FR) stages of ‘Kingsberry’ and ‘Sunnyberry’ cultivars. (DOCX)

S13 Table Metabolite contents of the ‘Kingsberry’ and ‘Sunnyberry’ cultivars. (DOCX)

S14 Table Statistics for analysis of variance for effect of cultivar, stage, and their interaction on selected gene expression. (DOCX)
