# Supplementary material for: Comparative transcriptome and metabolome analyses of two strawberry cultivars with different storability
Source: PLoS One. 2020 Dec 2;15(12):e0242556. doi: 10.1371/journal.pone.0242556 (PMC7710044; doi:10.1371/journal.pone.0242556)
Supplement: S1 Fig — (DOCX) [file pone.0242556.s001.docx]

**
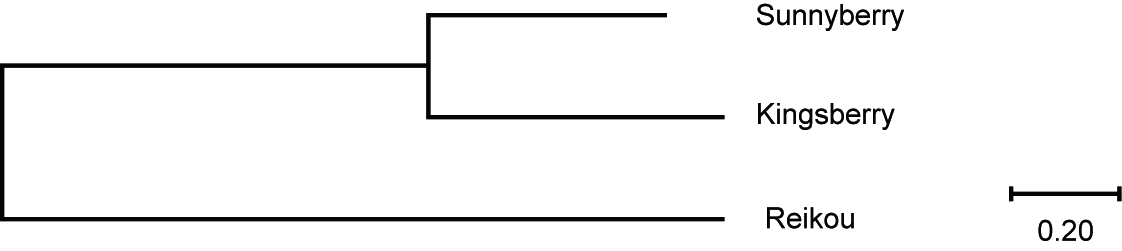
**

**S1 Fig. Phylogenetic relationships between ‘Sunnyberry’ and ‘Kingsberry’ cultivars and the reference cultivar ‘Reikou’ (strawberry-garden.kazusa.or.jp).** The neighbor-joining method was applied with MEGA X for 408,508 SNP positions. The bar indicates the number of substitutions per nucleotide position.
