# Supplementary material for: Comparative transcriptome and metabolome analyses of two strawberry cultivars with different storability
Source: PLoS One. 2020 Dec 2;15(12):e0242556. doi: 10.1371/journal.pone.0242556 (PMC7710044; doi:10.1371/journal.pone.0242556)
Supplement: S2 Fig — (DOCX) [file pone.0242556.s002.docx]

**
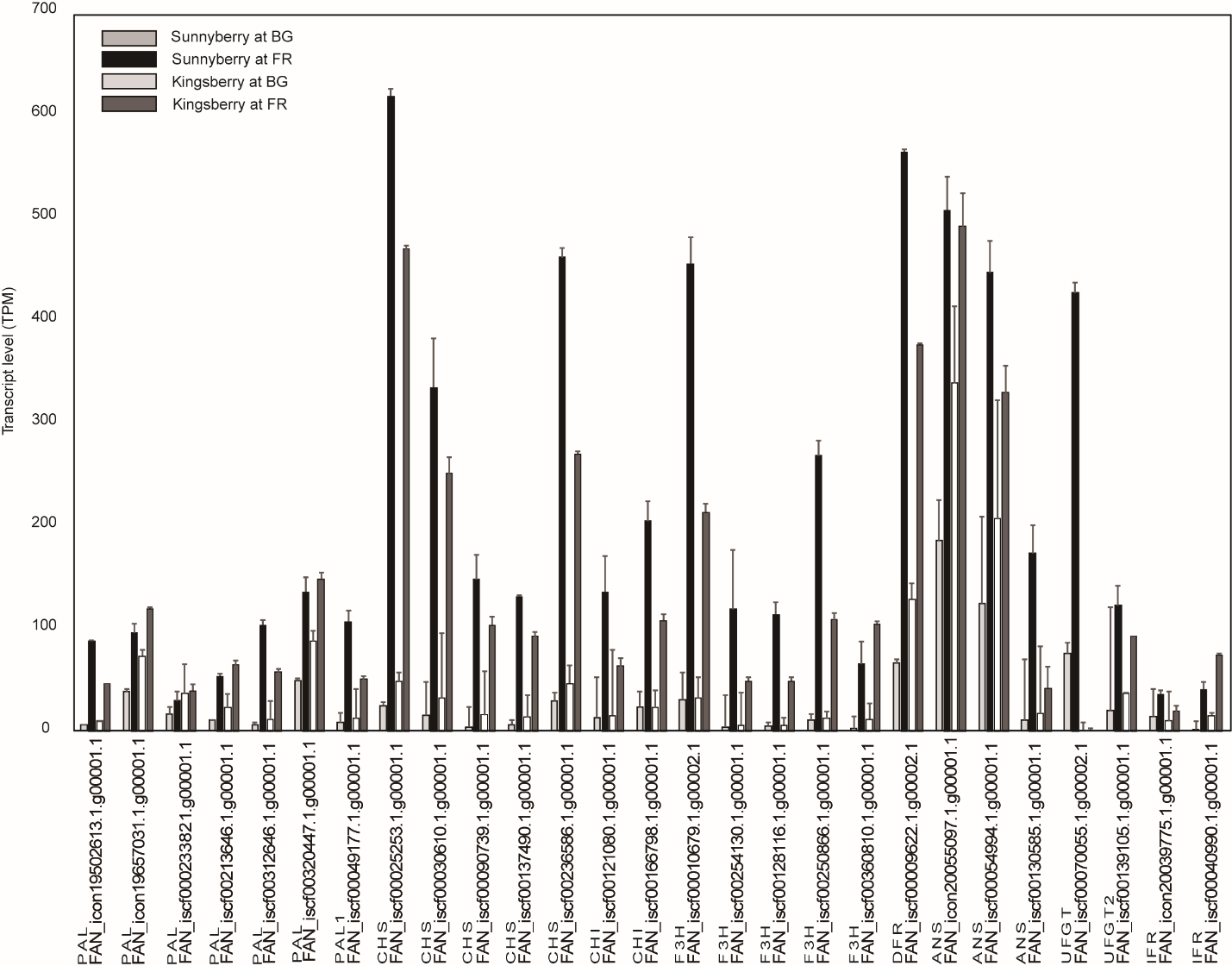
**

**S2 Fig. Transcript levels of differentially expressed genes in the anthocyanin-biosynthesis pathway.** ANS, anthocyanin synthase; CHI, chalcone isomerase; CHS, chalcone synthase; DFR, dihydroflavonol 4-reductase; F3H, flavanone 3-hydroxylase; IFR, isoflavone reductase; PAL, Phe ammonia-lyase; UFGT, UDP-glucoflavonoid 3-O-glucosyltransferase; BG, big-green stage; FR, full-red stage; TPM, transcripts per million.
