# Supplementary material for: Comparative transcriptome and metabolome analyses of two strawberry cultivars with different storability
Source: PLoS One. 2020 Dec 2;15(12):e0242556. doi: 10.1371/journal.pone.0242556 (PMC7710044; doi:10.1371/journal.pone.0242556)
Supplement: S3 Fig — (DOCX) [file pone.0242556.s003.docx]

**
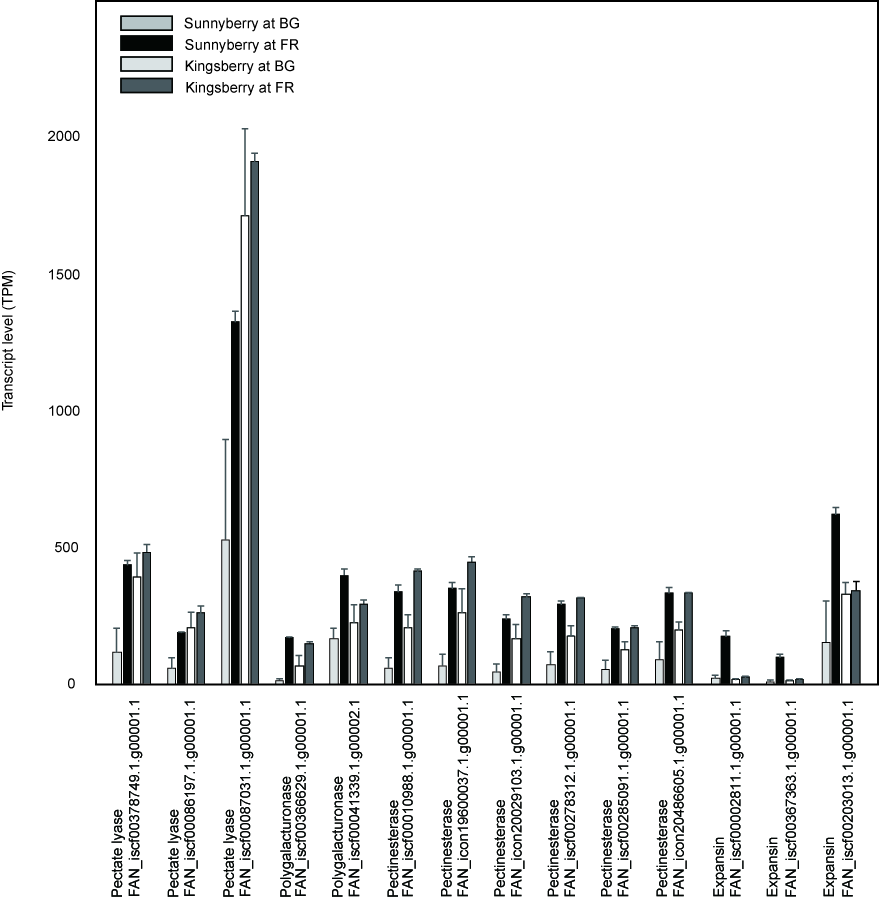
**

**S3 Fig. Transcript levels of differentially expressed genes related to cell-wall softening.** BG, big-green stage; FR, full-red stage; TPM, transcripts per million.
