# Supplementary material for: Comparative transcriptome and metabolome analyses of two strawberry cultivars with different storability
Source: PLoS One. 2020 Dec 2;15(12):e0242556. doi: 10.1371/journal.pone.0242556 (PMC7710044; doi:10.1371/journal.pone.0242556)
Supplement: S6 Fig — (DOCX) [file pone.0242556.s006.docx]

**
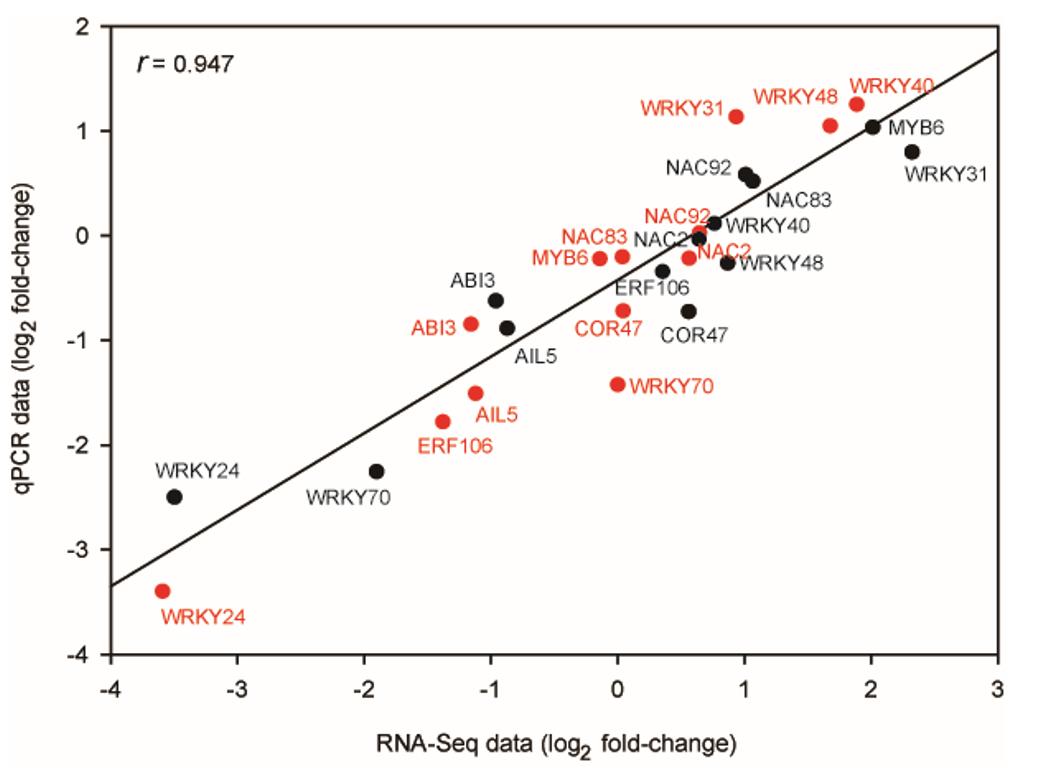
**

**S6 Fig. Correlation between RNA-Seq and qPCR data, based on log2 (fold-change) differences between ‘Kingsberry’ (red dots) and ‘Sunnyberry’ (black dots) cultivars at the big-green and fully-red stages.**
