# Supplementary material for: Comparative transcriptome and metabolome analyses of two strawberry cultivars with different storability
Source: PLoS One. 2020 Dec 2;15(12):e0242556. doi: 10.1371/journal.pone.0242556 (PMC7710044; doi:10.1371/journal.pone.0242556)
Supplement: S7 Fig — (DOCX) [file pone.0242556.s007.docx]

**
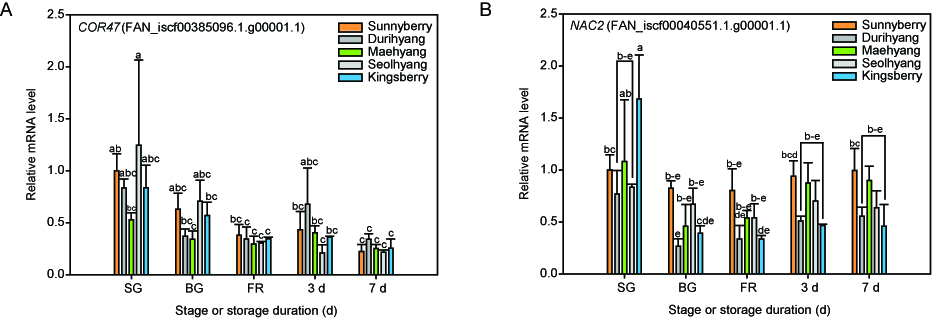
**

**S7 Fig. Relative-expression levels of (A) *COR47* and (B) *NAC2* in five different strawberry cultivars at the small-green (SG), big-green (BG), and fully-red (FR) developmental stages, or during storage at 10°C for up to 7 d.** Letters indicate significant differences at *P* < 0.05 in two-way ANOVA followed by the post-hoc Tukey’s HSD. Statistics can be found in **S10 Table.**
