# Supplementary material for: Comparative transcriptome and metabolome analyses of two strawberry cultivars with different storability
Source: PLoS One. 2020 Dec 2;15(12):e0242556. doi: 10.1371/journal.pone.0242556 (PMC7710044; doi:10.1371/journal.pone.0242556)
Supplement: S1 Table — (DOCX) [file pone.0242556.s008.docx]

**S1 Table. Crossing combination of five strawberry cultivars**

| **Cultivar** | **Crossing combination** | **Character** | **Registration year** |
| --- | --- | --- | --- |
| Durihyang | Sukhyang × Maehyang | Juicy | 2019 |
| Kingsberry | Akihime × NS00-13-09 | Big fruits | 2018 |
| Maehyang | Tochinomine × Akihime | Higher storability | 2014 |
| Seolhyang | Akihime × Red Pearl | A leading cultivar in Korea | 2014 |
| Sunnyberry | Sukhyang × Maehyang | Higher storability | 2019 |
