# Supplementary material for: Comparative transcriptome and metabolome analyses of two strawberry cultivars with different storability
Source: PLoS One. 2020 Dec 2;15(12):e0242556. doi: 10.1371/journal.pone.0242556 (PMC7710044; doi:10.1371/journal.pone.0242556)
Supplement: S2 Table — (DOCX) [file pone.0242556.s009.docx]

**S2 Table. Statistics for analysis of variance for effect of cultivar, storage duration, and their interaction on fruit firmness**

| Figure | Source | DF | Sum of squares^1)^ | Mean squares | F | Pr > F |
| --- | --- | --- | --- | --- | --- | --- |
| 1B | Model | 19 | 399.640 | 21.034 | 5.068 | < 0.0001 |
|  | storage duration | 3 | 53.059 | 17.686 | 4.261 | 0.007 |
|  | cultivar | 4 | 301.401 | 75.350 | 18.156 | < 0.0001 |
|  | storage duration × cultivar | 12 | 31.170 | 2.598 | 0.626 | 0.817 |
|  | Error | 138 | 572.736 | 4.150 |  |  |
|  | Corrected Total | 157 | 972.376 |  |  |  |
| 1C | Model | 19 | 0.733 | 0.039 | 28.974 | < 0.0001 |
|  | storage duration | 3 | 0.460 | 0.153 | 115.121 | < 0.0001 |
|  | cultivar | 4 | 0.092 | 0.023 | 17.209 | < 0.0001 |
|  | storage duration × cultivar | 12 | 0.182 | 0.015 | 11.359 | < 0.0001 |
|  | Error | 40 | 0.053 | 0.001 |  |  |
|  | Corrected Total | 59 | 0.786 |  |  |  |

^1)^Type III sum of squares analysis. Data for Fig 1B and C was used for two-way ANOVA. DF, degree of freedom; F, F value; Pr > F, the significance probability value associated with the F value.
