# Supplementary material for: Comparative transcriptome and metabolome analyses of two strawberry cultivars with different storability
Source: PLoS One. 2020 Dec 2;15(12):e0242556. doi: 10.1371/journal.pone.0242556 (PMC7710044; doi:10.1371/journal.pone.0242556)
Supplement: S3 Table — (DOCX) [file pone.0242556.s010.docx]

**S3 Table. Summary of RNA-Seq mapping**

| Sample name^1)^ | Read count  (pairs)^2)^ | Read length  (Mbp) | Q30 ratio  (%) | Q20 ratio  (%) | GC ratio  (%) | Mapped pairs (%) | Broken pairs (%) | Not mapped (%) |
| --- | --- | --- | --- | --- | --- | --- | --- | --- |
| KG_1 | 51,204,500 | 15,464 | 95.70 | 97.58 | 46.54 | 42.12 | 21.79 | 36.09 |
| KG_2 | 48,095,000 | 14,525 | 96.10 | 97.82 | 46.99 | 43.37 | 20.54 | 36.09 |
| KG_3 | 48,206,164 | 14,558 | 96.99 | 98.39 | 47.46 | 52.34 | 13.56 | 34.10 |
| KR_1 | 54,673,224 | 16,511 | 96.23 | 97.90 | 46.25 | 41.18 | 21.69 | 37.13 |
| KR_2 | 60,865,900 | 18,382 | 96.51 | 98.07 | 46.45 | 45.06 | 18.29 | 36.65 |
| KR_3 | 45,805,214 | 13,833 | 96.12 | 97.83 | 46.49 | 40.95 | 22.54 | 36.51 |
| SG_1 | 41,778,934 | 12,617 | 96.25 | 97.94 | 48.01 | 40.37 | 21.31 | 38.32 |
| SG_2 | 40,962,086 | 12,371 | 96.28 | 97.95 | 48.23 | 42.18 | 20.09 | 37.73 |
| SG_3 | 38,024,690 | 11,483 | 96.20 | 97.87 | 47.08 | 42.31 | 22.28 | 35.41 |
| SR_1 | 68,382,182 | 20,651 | 96.37 | 97.97 | 46.45 | 45.93 | 17.77 | 36.30 |
| SR_2 | 90,243,920 | 27,254 | 96.41 | 98.01 | 46.24 | 42.85 | 20.88 | 36.27 |
| SR_3 | 51,097,104 | 15,431 | 96.53 | 98.08 | 46.69 | 43.14 | 20.28 | 36.58 |

^1)^KG, ‘Kingsberry’ fruit at big-green stage; KR, ‘Kingsberry’ fruit at fully-red stage; SG, ‘Sunnyberry’ fruit at big-green stage; SR, ‘Sunnyberry’ fruit at fully-red stage ^2)^Numbers of qualified read pairs after trimming.
