# Supplementary material for: Comparative transcriptome and metabolome analyses of two strawberry cultivars with different storability
Source: PLoS One. 2020 Dec 2;15(12):e0242556. doi: 10.1371/journal.pone.0242556 (PMC7710044; doi:10.1371/journal.pone.0242556)
Supplement: S4 Table — (DOCX) [file pone.0242556.s011.docx]

**S4 Table.** **Sequences of primers used for qPCR analysis**

| **Gene** | **Gene ID** | **Forward primer (5'-3')** | | **Reverse primer (5'-3')** | | **Amplification efficiency (%)** | **Product size  (bp)** |
| --- | --- | --- | --- | --- | --- | --- | --- |
|  |  | **Sequence** | **Tm (°C)** | **Sequence** | **Tm (°C)** |  |  |
| *WRKY31* | FAN_iscf00182457.1.g00001.1 | GCAACTCTCAGTCAACCTC | 55.6 | CTTGTTGTTGTTATCGGCG | 55.2 | 91.3 | 133 |
| *WRKY40* | FAN_iscf00264237.1.g00001.1 | AGGTTCAAAGAAGTGTCGAG | 55.3 | GATGCTTCGATTTGAGAAGG | 54.5 | 93.1 | 91 |
| *WRKY48* | FAN_iscf00018791.1.g00001.1 | GGCTGGTTATGGTGTAGTTC | 55.8 | TAACGTTCAAAGAAGGCGAA | 55.6 | 116.0 | 104 |
| *WRKY70* | FAN_iscf00010757.1.g00002.1 | GATCATGGTGGTCATCTCTC | 55.0 | TATCGTTAAGGTTGTCCGTC | 54.9 | 98.7 | 118 |
| *MYB6* | FAN_iscf00125059.1.g00001.1 | TGTTCGAGTCCAAAACTCC | 55.1 | TAGAAACCCTGAGATGGACA | 55.2 | 93.6 | 147 |
| *ABI3* | FAN_iscf00351130.1.g00001.1 | GAATCAAATGCTGGAGACTG | 54.4 | GTTGTTGTTGATGCTGACTC | 55.2 | 111.0 | 122 |
| *ERF106* | FAN_iscf00004671.1.g00001.1 | GTTTGACACTGATGTGGATG | 54.6 | GTATTACTCGGCGGGTTAG | 54.9 | 110.7 | 120 |
| *AIL5* | FAN_iscf00193262.1.g00001.1 | AGTGGATTTTCAAGAGGAGC | 55.3 | AGGTACAGATCTTTGTTGCC | 55.4 | 108.3 | 110 |
| *NAC83* | FAN_iscf00089695.1.g00001.1 | AACCAAGGATTACTCGACCT | 55.9 | CGCTAGATACGACATCTGTG | 55.6 | 90.4 | 120 |
| *NAC92* | FAN_iscf00019465.1.g00002.1 | ATCAAGGCTCCTCCTTTAAC | 54.7 | CACTGTCTGCTATCTAAGCC | 55.4 | 109.9 | 145 |
| *COR47* | FAN_iscf00385096.1.g00001.1 | GGGTTTCCTTGAGAAGATCA | 54.7 | CAACATTTTCATACTCCGGC | 55.1 | 90.1 | 98 |
| *NAC2* | FAN_iscf00040551.1.g00001.1 | TCTCGGTTCCGATAATTGC | 55.1 | TGAACCGTTTGGGTATTTCC | 55.9 | 111.9 | 131 |
| *GAPDH* | AB363963.1 | CCTGCTCTCAATGGCAAATT | 56.7 | GATCTCTTCATCTTTCCCTCA | 54.5 | 91.5 | 155 |

qPCR, quantitative polymerase chain reaction; Tm, melting temperature.
