# Supplementary material for: Comparative transcriptome and metabolome analyses of two strawberry cultivars with different storability
Source: PLoS One. 2020 Dec 2;15(12):e0242556. doi: 10.1371/journal.pone.0242556 (PMC7710044; doi:10.1371/journal.pone.0242556)
Supplement: S5 Table — (DOCX) [file pone.0242556.s012.docx]

**S5 Table.** **Information for the polar metabolites annotated in the strawberry fruit using GC-MS**

|  | **Common name** | **Derivate** | **CAS** | **Retention time  (min)** | **m/z** | **identification  level** | **R. match** |
| --- | --- | --- | --- | --- | --- | --- | --- |
| 1 | acetic acid | 2tms | 33581-77-0 | 18.65 | 147 | 2 | 739 |
| 2 | arabinose | 4tms | 9601869 (PubChem) | 32.24 | 147 | 2 | 876 |
| 3 | citric acid | 4tms | 14330-97-3 | 35.83 | 147 | 2 | 913 |
| 4 | fructofuranose | 5tms | 380166 (NIST) | 35.67 | 217 | 2 | 803 |
| 5 | fructose | 5tms | 380189 (NIST) | 36.94 | 103 | 2 | 950 |
| 6 | fructose | 5tms | 380190 (NIST) | 37.10 | 103 | 2 | 936 |
| 7 | galactopyranoside | 4tms | 4133-45-3 | 39.97 | 204 | 2 | 731 |
| 8 | galactose | 5tms | 128705-71-5 | 37.88 | 147 | 2 | 936 |
| 9 | gluconic acid | 4tms | 55515-33-8 | 38.63 | 217 | 2 | 816 |
| 10 | glucopyranose | 5tms | 19126-99-9 | 39.01 | 204 | 2 | 820 |
| 11 | glucose | 5tms | 130405-10-6 | 37.44 | 147 | 2 | 962 |
| 12 | lactic acid | 2tms | 17596-96-2 | 16.14 | 147 | 2 | 797 |
| 13 | malic acid | 3tms | 65143-63-7 | 28.24 | 147 | 2 | 932 |
| 14 | myo-inositol | 6tms | 2582-79-8 | 41.28 | 147 | 2 | 888 |
| 15 | oxoglutaric acid | 2tms | 1027138-06-2 | 36.48 | 157 | 2 | 748 |
| 16 | quinic acid | 5tms | NA | 36.66 | 345 | 2 | 893 |
| 17 | ribitol | 5tms | 32381-53-6 | 33.79 | 147 | 2 | 914 |
| 18 | succinic acid | 2tms | 40309-57-7 | 23.67 | 75 | 2 | 885 |
| 19 | sucrose | 8tms | 19159-25-2 | 50.27 | 361 | 2 | 934 |
| 20 | tagatofuranose | 5tms | 380125 (NIST) | 35.5 | 217 | 2 | 833 |
| 21 | threonic acid | 4tms | NA | 29.95 | 147 | 2 | 808 |
| 22 | xylose | 4tms | 56196-07-7 | 32.05 | 103 | 2 | 883 |
| 23 | unknown1 | NA | NA | 21.87 | 42 | 4 | NA |
| 24 | unknown2 | NA | NA | 40.42 | 75 | 4 | NA |
| 25 | unknown3 | NA | NA | 21.65 | 73 | 4 | NA |
| 26 | unknown4 | NA | NA | 23.58 | 73 | 4 | NA |
| 27 | unknown5 | NA | NA | 24.07 | 147 | 4 | NA |

GC-MS, gas chromatography-mass spectrometry; CAS, chemical abstract service.
