# Supplementary material for: Comparative transcriptome and metabolome analyses of two strawberry cultivars with different storability
Source: PLoS One. 2020 Dec 2;15(12):e0242556. doi: 10.1371/journal.pone.0242556 (PMC7710044; doi:10.1371/journal.pone.0242556)
Supplement: S10 Table — (DOCX) [file pone.0242556.s017.docx]

**S10 Table. Most highly expressed transcripts (TPM > 1000 in one of the cultivars) in ‘Kingsberry’ and ‘Sunnyberry’ during the big-green (BG) stage**

| **Gene ID** | **Sequence Description** | **FC KG vs. SG^1)^** | ***P* value** | **FDR *P* value** | **KG TPM Mean** | **KGTPM SD** | **SG TPM Mean** | **SG TPM SD** |
| --- | --- | --- | --- | --- | --- | --- | --- | --- |
| FAN_iscf00098624.1.g00003.1 | legumin type B-like | 2.80 | 2.77E-05 | 2.17E-03 | 4505.7 | 708.6 | 11142.6 | 3172.8 |
| FAN_iscf00252733.1.g00001.1 | transmembrane protein | 6.17 | 6.27E-11 | 2.79E-08 | 4202.0 | 1303.4 | 22762.9 | 11514.0 |
| FAN_iscf00254621.1.g00001.1 | legumin type B-like | 1.46 | 0.125 | 0.959 | 4094.3 | 643.3 | 5295.8 | 1431.2 |
| FAN_iscf00016921.1.g00001.1 | putative 11-S seed storage protein, | 2.61 | 5.15E-06 | 5.37E-04 | 3199.9 | 259.6 | 7434.2 | 1461.3 |
| FAN_iscf00154794.1.g00001.1 | edestin 1 | 2.59 | 9.85E-05 | 6.14E-03 | 2623.4 | 120.0 | 5988.1 | 2053.6 |
| FAN_iscf00225504.1.g00001.1 | legumin A-like | 2.78 | 2.09E-05 | 1.73E-03 | 2502.0 | 381.1 | 6152.1 | 1787.2 |
| FAN_iscf00033383.1.g00001.1 | 14 kDa proline-rich protein DC2.15-like | -1.48 | 0.026 | 0.384 | 2498.7 | 249.3 | 1532.5 | 314.7 |
| FAN_iscf00072314.1.g00002.1 | expansin-A8 | -4.45 | 1.46E-06 | 1.81E-04 | 2404.7 | 238.3 | 510.0 | 450.4 |
| FAN_iscf00258503.1.g00001.1 | endoglucanase 6 | -1.35 | 0.169 | 1.000 | 2115.6 | 161.4 | 1443.8 | 726.0 |
| FAN_iscf00012988.1.g00001.1 | vinorine synthase-like | -7.57 | 3.11E-07 | 4.82E-05 | 1912.5 | 929.7 | 241.6 | 251.7 |
| FAN_iscf00266670.1.g00001.1 | legumin A-like | 3.57 | 1.15E-09 | 3.73E-07 | 1872.0 | 272.1 | 5946.9 | 1330.1 |
| FAN_iscf00093676.1.g00001.1 | 14 kDa proline-rich protein DC2.15-like | -1.91 | 1.07E-04 | 6.54E-03 | 1865.9 | 136.6 | 885.3 | 150.3 |
| FAN_iscf00015241.1.g00001.1 | elongation factor 1-α | -1.12 | 0.472 | 1.000 | 1731.7 | 114.6 | 1395.3 | 104.4 |
| FAN_iscf00087031.1.g00001.1 | probable pectate lyase 8 | -3.04 | 3.67E-05 | 2.76E-03 | 1718.1 | 316.5 | 531.1 | 368.2 |
| FAN_iscf00075557.1.g00001.1 | putative 11-S seed storage protein | 2.36 | 3.29E-04 | 0.016 | 1693.9 | 229.7 | 3533.8 | 1220.8 |
| FAN_iscf00034497.1.g00001.1 | uncharacterized protein LOC108867880 | 3.33 | 4.10E-07 | 6.12E-05 | 1523.5 | 293.8 | 4477.7 | 1763.0 |
| FAN_iscf00126194.1.g00001.1 | probable pectate lyase 8 | -1.62 | 0.022 | 0.349 | 1516.7 | 206.7 | 866.8 | 369.2 |
| FAN_iscf00151061.1.g00002.1 | vicilin-like antimicrobial peptides 2-2 | 2.49 | 1.05E-04 | 6.49E-03 | 1485.9 | 177.8 | 3267.9 | 1059.5 |
| FAN_iscf00162896.1.g00001.1 | legumin A-like | 2.12 | 1.58E-03 | 0.054 | 1482.8 | 62.0 | 2784.6 | 801.2 |
| FAN_iscf00209593.1.g00001.1 | polyubiquitin 11 | -1.08 | 0.661 | 1.000 | 1448.0 | 58.3 | 1224.0 | 247.7 |
| FAN_iscf00115557.1.g00001.1 | legumin A-like | 1.76 | 7.59E-03 | 0.170 | 1420.8 | 122.6 | 2230.7 | 407.4 |
| FAN_icon20485241.1.g00001.1 | legumin A-like | 3.91 | 9.48E-07 | 1.26E-04 | 1419.4 | 220.6 | 4871.2 | 2234.4 |
| FAN_iscf00189022.1.g00001.1 | legumin type B-like | 1.67 | 0.010 | 0.211 | 1351.2 | 127.5 | 2018.4 | 379.3 |
| FAN_icon20792886.1.g00001.1 | vinorine synthase-like | -6.26 | 7.10E-08 | 1.36E-05 | 1300.2 | 388.5 | 197.3 | 191.9 |
| FAN_iscf00117578.1.g00002.1 | polyubiquitin | 1.01 | 0.945 | 1.000 | 1243.7 | 57.6 | 1146.3 | 258.4 |
| FAN_iscf00050777.1.g00009.1 | sucrose-binding protein | 1.40 | 0.095 | 0.838 | 1228.4 | 124.4 | 1534.9 | 293.2 |
| FAN_iscf00221098.1.g00001.1 | peroxiredoxin-2B | 1.00 | 0.996 | 1.000 | 1224.0 | 59.7 | 1114.3 | 188.4 |
| FAN_iscf00283840.1.g00002.1 | auxin-responsive protein SAUR72-like | -2.01 | 2.30E-03 | 0.072 | 1158.7 | 252.4 | 535.9 | 269.2 |
| FAN_icon20281967.1.g00001.1 | legumin type B-like | 1.94 | 4.68E-03 | 0.120 | 1039.0 | 112.1 | 1791.2 | 466.3 |
| FAN_iscf00134048.1.g00001.1 | cinnamate beta-D-glucosyltransferase-like | -6.76 | 3.36E-10 | 1.26E-07 | 1011.4 | 309.1 | 141.5 | 119.1 |
| FAN_iscf00239419.1.g00001.1 | NA | 2.42 | 9.12E-05 | 5.77E-03 | 1003.6 | 134.0 | 2145.2 | 641.1 |
| FAN_iscf00264933.1.g00001.1 | legumin A-like | 4.63 | 2.32E-09 | 6.87E-07 | 937.3 | 183.6 | 3802.2 | 1651.6 |
| FAN_iscf00045844.1.g00003.1 | dormancy-associated protein homolog 3 isoform X1 | 1.54 | 0.040 | 0.508 | 920.9 | 117.1 | 1257.0 | 401.5 |
| FAN_icon19742839.1.g00001.1 | legumin A-like | 3.95 | 3.34E-07 | 5.14E-05 | 724.1 | 60.9 | 2512.7 | 1114.1 |
| FAN_iscf00254556.1.g00003.1 | 21 kDa protein-like | 1.68 | 2.34E-03 | 0.073 | 675.3 | 102.1 | 1022.0 | 25.6 |
| FAN_iscf00300126.1.g00001.1 | legumin A-like | 1.84 | 1.80E-03 | 0.060 | 663.6 | 81.6 | 1087.4 | 214.7 |
| FAN_iscf00343817.1.g00001.1 | embryogenic cell protein 40 | 2.14 | 5.68E-04 | 0.025 | 636.7 | 20.2 | 1208.6 | 323.9 |
| FAN_iscf00161038.1.g00001.1 | edestin 1 | 4.37 | 8.99E-09 | 2.28E-06 | 585.7 | 50.2 | 2245.5 | 993.7 |
| FAN_iscf00138842.1.g00002.1 | major latex allergen Hev b 5-like | 2.92 | 8.85E-08 | 1.66E-05 | 558.1 | 180.3 | 1458.1 | 37.8 |
| FAN_iscf00016631.1.g00001.1 | senescence-associated protein | 4.72 | 3.54E-09 | 9.95E-07 | 278.9 | 96.6 | 1159.0 | 523.1 |

^1)^KG, ‘Kingsberry’ fruit at BG; SG, ‘Sunnyberry’ fruit at BG; FC, fold-change; FDR, false discovery rate; TPM, transcripts per million; SD, standard deviation.
