# Supplementary material for: Comparative transcriptome and metabolome analyses of two strawberry cultivars with different storability
Source: PLoS One. 2020 Dec 2;15(12):e0242556. doi: 10.1371/journal.pone.0242556 (PMC7710044; doi:10.1371/journal.pone.0242556)
Supplement: S11 Table — (DOCX) [file pone.0242556.s018.docx]

**S11 Table.** **Most highly expressed transcripts (TPM > 1000 in one of the cultivars) in ‘Kingsberry’ and ‘Sunnyberry’ during the fully-red (FR) stage**

| **Gene ID** | **Sequence Description** | **FC KR vs. SR^1)^** | ***P* value** | **FDR *P* value** | **KR TPM Mean** | **KR TPM SD** | **SR TPM mean** | **SR TPM SD** |
| --- | --- | --- | --- | --- | --- | --- | --- | --- |
| FAN_iscf00012988.1.g00001.1 | vinorine synthase-like | -1.11 | 0.787 | 1.000 | 6033.0 | 295.4 | 5008.9 | 154.5 |
| FAN_icon20792886.1.g00001.1 | vinorine synthase-like | -1.24 | 0.529 | 1.000 | 4502.7 | 212.2 | 3352.3 | 162.5 |
| FAN_iscf00033383.1.g00001.1 | 14 kDa proline-rich protein DC2.15-like | 1.08 | 0.673 | 1.000 | 3237.3 | 184.7 | 3221.1 | 383.8 |
| FAN_iscf00252733.1.g00001.1 | transmembrane protein, putative | 1.37 | 0.258 | 1.000 | 3116.1 | 259.7 | 3947.8 | 966.3 |
| FAN_icon20484069.1.g00001.1 | vinorine synthase-like | -1.66 | 0.100 | 0.903 | 2420.0 | 125.7 | 1342.6 | 32.7 |
| FAN_iscf00134048.1.g00001.1 | cinnamate beta-D-glucosyltransferase-like | -1.26 | 0.453 | 1.000 | 2048.3 | 83.0 | 1502.5 | 94.0 |
| FAN_iscf00093676.1.g00001.1 | 14 kDa proline-rich protein DC2.15-like | -1.06 | 0.718 | 1.000 | 1949.0 | 33.6 | 1694.1 | 82.6 |
| FAN_iscf00072314.1.g00002.1 | expansin-A8 | -1.69 | 0.090 | 0.846 | 1942.8 | 80.2 | 1062.8 | 97.6 |
| FAN_iscf00087031.1.g00001.1 | probable pectate lyase 8 | -1.34 | 0.267 | 1.000 | 1932.5 | 39.3 | 1326.7 | 56.5 |
| FAN_iscf00050333.1.g00001.1 | 2-methylene-furan-3-one reductase | 2.34 | 0.009 | 0.194 | 1729.6 | 97.8 | 3731.2 | 161.7 |
| FAN_icon20826016.1.g00001.1 | non-functional NADPH-dependent codeinone reductase 2-like | 2.08 | 0.009 | 0.194 | 1696.2 | 56.1 | 3259.0 | 179.9 |
| FAN_iscf00105939.1.g00001.1 | UDP-glucose glucosyltransferase | -1.19 | 0.560 | 1.000 | 1667.1 | 81.3 | 1292.5 | 4.3 |
| FAN_iscf00015241.1.g00001.1 | elongation factor 1-alpha | 1.02 | 0.881 | 1.000 | 1663.9 | 39.2 | 1571.1 | 63.8 |
| FAN_iscf00209415.1.g00002.1 | 2-methylene-furan-3-one reductase-like | 1.84 | 0.034 | 0.483 | 1621.5 | 72.1 | 2752.9 | 157.7 |
| FAN_iscf00144148.1.g00001.1 | methanol O-anthraniloyltransferase-like | -1.24 | 0.503 | 1.000 | 1595.1 | 132.6 | 1183.6 | 78.0 |
| FAN_iscf00034497.1.g00001.1 | PREDICTED: uncharacterized protein LOC108867880 | -1.00 | 1.000 | 1.000 | 1467.3 | 42.9 | 1356.5 | 308.4 |
| FAN_iscf00283840.1.g00002.1 | auxin-responsive protein SAUR72-like | 1.25 | 0.332 | 1.000 | 1448.0 | 77.0 | 1669.4 | 99.5 |
| FAN_iscf00004625.1.g00002.1 | codeine O-demethylase-like | 1.16 | 0.725 | 1.000 | 1442.6 | 79.2 | 1544.1 | 115.0 |
| FAN_iscf00258503.1.g00001.1 | endoglucanase 6 | 1.16 | 0.495 | 1.000 | 1383.4 | 96.3 | 1482.4 | 187.7 |
| FAN_iscf00079376.1.g00001.1 | exopolygalacturonase-like | 2.20 | 0.003 | 0.083 | 1378.9 | 45.7 | 2800.4 | 222.4 |
| FAN_iscf00126194.1.g00001.1 | probable pectate lyase 8 | -1.06 | 0.765 | 1.000 | 1357.6 | 25.5 | 1176.6 | 21.3 |
| FAN_iscf00305385.1.g00001.1 | exopolygalacturonase-like | 1.87 | 0.017 | 0.302 | 1253.2 | 76.4 | 2161.0 | 108.4 |
| FAN_iscf00183386.1.g00001.1 | acyl carrier protein 1, chloroplastic-like | 1.08 | 0.748 | 1.000 | 1149.7 | 19.5 | 1145.7 | 44.8 |
| FAN_iscf00231729.1.g00001.1 | probable inactive 2-oxoglutarate-dependent dioxygenase AOP2 | 1.61 | 0.012 | 0.238 | 1117.3 | 25.6 | 1662.9 | 51.9 |
| FAN_iscf00209593.1.g00001.1 | polyubiquitin 11 | 1.41 | 0.043 | 0.556 | 1072.4 | 30.4 | 1393.5 | 104.5 |
| FAN_iscf00271282.1.g00001.1 | pyruvate decarboxylase 2 | 1.21 | 0.437 | 1.000 | 975.9 | 32.7 | 1084.7 | 104.7 |
| FAN_iscf00010083.1.g00003.1 | endoglucanase 8 | 1.52 | 0.150 | 1.000 | 956.3 | 72.9 | 1344.8 | 14.8 |
| FAN_iscf00058042.1.g00001.1 | cysteine protease RD19A-like | 1.25 | 0.173 | 1.000 | 955.0 | 37.1 | 1099.4 | 37.8 |
| FAN_iscf00117578.1.g00002.1 | polyubiquitin | 1.49 | 0.016 | 0.300 | 938.7 | 34.7 | 1293.6 | 61.1 |
| FAN_iscf00036526.1.g00001.1 | caffeic acid 3-O-methyltransferase | 2.12 | 0.003 | 0.089 | 936.7 | 87.7 | 1831.2 | 68.4 |
| FAN_iscf00050777.1.g00009.1 | sucrose-binding protein-like | 1.82 | 0.003 | 0.088 | 767.5 | 218.8 | 1284.8 | 136.0 |
| FAN_iscf00016921.1.g00001.1 | putative 11-S seed storage protein, plant | 2.96 | 2E-07 | 3E-05 | 758.4 | 254.8 | 2069.6 | 255.2 |
| FAN_iscf00266670.1.g00001.1 | legumin A-like | 3.75 | 4E-10 | 9E-08 | 354.3 | 109.6 | 1225.4 | 159.7 |
| FAN_iscf00138842.1.g00002.1 | major latex allergen Hev b 5-like | 6.88 | 0 | 0 | 186.0 | 42.9 | 1179.2 | 37.3 |

^1)^KR, ‘Kingsberry’ fruit at FR; SR, ‘Sunnyberry’ fruit at FR; FC, fold-change; FDR, false discovery rate; TPM, transcripts per million; SD, standard deviation.
