# Supplementary material for: Comparative transcriptome and metabolome analyses of two strawberry cultivars with different storability
Source: PLoS One. 2020 Dec 2;15(12):e0242556. doi: 10.1371/journal.pone.0242556 (PMC7710044; doi:10.1371/journal.pone.0242556)
Supplement: S12 Table — (DOCX) [file pone.0242556.s019.docx]

**S12 Table.** **Metabolite accumulation between big-green (BG) and fully-red (FR) stages of ‘Kingsberry’ and ‘Sunnyberry’ cultivars**

| **Category** | **Metabolite** | **KG vs. KR^1)^** | | **SG vs. SR^2)^** | |
| --- | --- | --- | --- | --- | --- |
|  |  | **log_2_ fold-change** | ***P* value** | **log_2_ fold-change** | ***P* value** |
| Sugars | arabinose | 0.41 | 0.28 | 1.15 | 0.01 |
|  | fructofuranose | 0.86 | 0.22 | 1.29 | 0.49 |
|  | fructose | -0.03 | 0.89 | 0.12 | 0.50 |
|  | galactopyranoside | 0.56 | 0.17 | 0.09 | 0.85 |
|  | galactose | -0.05 | 0.84 | 0.14 | 0.46 |
|  | glucopyranose | 0.71 | 0.06 | 0.81 | 0.39 |
|  | glucose | -0.04 | 0.86 | 0.17 | 0.39 |
|  | sucrose | 0.75 | 0.23 | 1.84 | 0.01 |
|  | tagatofuranose | 1.28 | 0.42 | 1.56 | 0.38 |
|  | xylose | -0.61 | 0.47 | 1.03 | 0.01 |
| Organic acids | acetic acid | -0.78 | 0.11 | 0.20 | 0.53 |
|  | citric acid | -0.77 | 0.12 | -0.98 | 0.04 |
|  | gluconic acid | -0.33 | 0.46 | 0.49 | 0.28 |
|  | malic acid | 0.06 | 0.84 | 0.69 | 0.04 |
|  | oxoglutaric acid | -0.32 | 0.31 | 0.58 | 0.06 |
|  | quinic acid | -0.06 | 0.83 | 0.10 | 0.32 |
|  | succinic acid | 0.12 | 0.69 | 1.29 | 0.01 |
| Amino acids | alanine | 0.60 | 0.32 | 1.98 | 0.01 |
|  | arginine | -2.14 | 0.00 | -1.55 | 0.00 |
|  | asparagine | -0.31 | 0.32 | -0.05 | 0.64 |
|  | aspartic acid | -0.21 | 0.06 | -0.11 | 0.49 |
|  | GABA | 0.34 | 0.37 | 0.02 | 0.95 |
|  | glutamic acid | -0.36 | 0.15 | -0.43 | 0.13 |
|  | glutamine | -0.17 | 0.66 | 1.40 | 0.01 |
|  | glycine | 0.61 | 0.11 | 0.67 | 0.05 |
|  | histidine | -0.65 | 0.15 | 0.35 | 0.49 |
|  | isoleucine | -0.42 | 0.09 | -0.07 | 0.77 |
|  | leucine | 0.41 | 0.18 | 2.02 | 0.00 |
|  | lysine | -0.87 | 0.17 | -1.37 | 0.02 |
|  | methionine | 0.16 | 0.77 | 0.99 | 0.02 |
|  | phenylalanine | -0.37 | 0.18 | -0.32 | 0.18 |
|  | proline | 0.09 | 0.87 | 0.57 | 0.32 |
|  | serine | 0.64 | 0.16 | 1.40 | 0.00 |
|  | threonine | 0.21 | 0.29 | 0.58 | 0.09 |
|  | tryptophan | 1.66 | 0.02 | 2.32 | 0.02 |
|  | tyrosine | 0.76 | 0.03 | 0.62 | 0.11 |
|  | valine | -0.36 | 0.28 | 0.42 | 0.06 |
| Fatty acids | alpha-linolenic acid | -0.54 | 0.20 | -0.22 | 0.46 |
|  | arachidic acid | -0.92 | 0.01 | -0.21 | 0.58 |
|  | dodecanoic acid | 0.15 | 0.11 | 0.61 | 0.04 |
|  | linoleic acid | -0.50 | 0.10 | -0.07 | 0.85 |
|  | oleic acid | -0.54 | 0.12 | 0.28 | 0.61 |
|  | palmitic acid | -0.45 | 0.01 | -0.32 | 0.00 |
| Others | lactic acid | 0.19 | 0.60 | 0.34 | 0.36 |
|  | myo-inositol | 0.43 | 0.22 | 0.66 | 0.37 |
|  | threonic acid | -1.49 | 0.04 | -1.10 | 0.01 |

^1)^KG, ‘Kingsberry’ fruit at BG; KR, ‘Kingsberry’ fruit at FR; ^2)^SG, ‘Sunnyberry’ fruit at BG; SR, ‘Sunnyberry’ fruit at FR
