# Supplementary material for: Comparative transcriptome and metabolome analyses of two strawberry cultivars with different storability
Source: PLoS One. 2020 Dec 2;15(12):e0242556. doi: 10.1371/journal.pone.0242556 (PMC7710044; doi:10.1371/journal.pone.0242556)
Supplement: S13 Table — (DOCX) [file pone.0242556.s020.docx]

**S13 Table. Metabolite content of the ‘Kingsberry’ and ‘Sunnyberry’ cultivars**

| **Category** | **Metabolite** | **Metabolite content (mean ± SD)** | | | | |
| --- | --- | --- | --- | --- | --- | --- |
|  |  | **‘Kingsberry’ at BG^1)^** | **‘Kingsberry’ at FR^2)^** | **‘Sunnyberry’ at BG** | **‘Sunnyberry’ at FR** |  |
| Sugars (mg kg^−1^  fresh weight) | arabinose | 158.48 ± 50.14 | 211.02 ± 53.93 | 111.88 ± 17.54 | 247.60 ± 45.41 |  |
|  | fructofuranose | 6.00 ± 0.69 | 7.25 ± 1.76 | 6.49 ± 1.09 | 23.75 ± 19.61 |  |
|  | fructose (mg g^−1^) | 19.36 ± 2.31 | 18.96 ± 4.08 | 20.16 ± 1.79 | 21.85 ± 3.50 |  |
|  | galactopyranoside | 33.95 ± 6.30 | 50.10 ± 15.26 | 45.39 ± 19.61 | 48.48 ± 19.10 |  |
|  | galactose (mg g^−1^) | 2.33 ± 0.29 | 2.25 ± 0.54 | 2.24 ± 0.16 | 2.47 ± 0.44 |  |
|  | glucopyranose | 31.99 ± 5.50 | 52.47 ± 12.88 | 42.38 ± 21.89 | 74.23 ± 53.38 |  |
|  | glucose (mg g^−1^) | 15.25 ± 1.65 | 14.85 ± 3.26 | 14.91 ± 1.20 | 16.79 ± 3.13 |  |
|  | sucrose (mg g^−1^) | 6.71 ± 2.67 | 11.24 ± 4.79 | 3.07 ± 0.72 | 11.01 ± 2.50 |  |
|  | tagatofuranose | 2.22 ± 3.85 | 8.07 ± 1.46 | 6.55 ± 1.55 | 19.40 ± 13.40 |  |
|  | xylose | 65.43 ± 48.54 | 42.78 ± 11.06 | 24.53 ± 2.96 | 49.99 ± 8.10 |  |
| Organic acids (mg kg^−1^  fresh weight) | acetic acid | 37.83 ± 11.84 | 21.97 ± 6.35 | 24.87 ± 8.21 | 28.63 ± 4.97 |  |
|  | citric acid (mg g^−1^) | 4.44 ± 0.72 | 2.61 ± 1.46 | 3.24 ± 0.86 | 1.65 ± 0.37 |  |
|  | gluconic acid | 41.11 ± 2.92 | 32.63 ± 17.88 | 31.24 ± 17.35 | 44.02 ± 3.98 |  |
|  | malic acid (mg g^−1^) | 0.92 ± 0.14 | 0.96 ± 0.32 | 1.01 ± 0.19 | 1.62 ± 0.31 |  |
|  | oxoglutaric acid | 95.95 ± 5.29 | 76.83 ± 28.21 | 59.3 ± 14.04 | 88.63 ± 13.23 |  |
|  | quinic acid | 29.46 ± 4.29 | 28.30 ± 7.74 | 29.78 ± 5.73 | 31.90 ± 4.56 |  |
|  | succinic acid | 16.56 ± 3.34 | 17.95 ± 4.56 | 9.27 ± 3.40 | 22.61 ± 1.87 |  |
| Amino  acids (mg kg^−1^  fresh weight) | alanine | 95.74 ± 37.92 | 144.75 ± 65.18 | 21.03 ± 7.33 | 82.97 ± 19.54 |  |
|  | arginine | 12.15 ± 2.18 | 2.76 ± 0.88 | 19.00 ± 2.04 | 6.49 ± 0.46 |  |
|  | asparagine | 561.81 ± 113.46 | 452.84 ± 121.91 | 752.85 ± 83.57 | 727.63 ± 24.54 |  |
|  | aspartic acid | 127.68 ± 5.76 | 110.64 ± 9.52 | 107.14 ± 14.67 | 99.19 ± 10.39 |  |
|  | GABA | 6.95 ± 2.66 | 8.81 ± 1.80 | 2.55 ± 0.81 | 2.59 ± 0.65 |  |
|  | glutamic acid | 188.16 ± 37.73 | 146.66 ± 15.82 | 196.21 ± 42.93 | 145.85 ± 17.02 |  |
|  | glutamine | 512.26 ± 73.78 | 454.37 ± 196.56 | 109.94 ± 39.16 | 289.77 ± 39.87 |  |
|  | glycine | 3.43 ± 0.25 | 5.21 ± 1.46 | 2.95 ± 1.06 | 4.69 ± 0.35 |  |
|  | histidine | 3.91 ± 1.10 | 2.50 ± 0.09 | 2.48 ± 0.82 | 3.16 ± 1.34 |  |
|  | isoleucine | 3.94 ± 0.46 | 2.95 ± 0.63 | 3.96 ± 0.88 | 3.77 ± 0.62 |  |
|  | leucine | 3.50 ± 0.72 | 4.64 ± 0.97 | 2.90 ± 0.54 | 11.77 ± 1.92 |  |
|  | lysine | 0.72 ± 0.25 | 0.39 ± 0.22 | 2.12 ± 0.47 | 0.82 ± 0.39 |  |
|  | methionine | 0.86 ± 0.26 | 0.96 ± 0.50 | 0.44 ± 0.07 | 0.88 ± 0.20 |  |
|  | phenylalanine | 7.10 ± 0.91 | 5.50 ± 1.43 | 5.26 ± 0.87 | 4.22 ± 0.69 |  |
|  | proline | 55.31 ± 14.76 | 59.03 ± 34.45 | 21.35 ± 15.63 | 31.61 ± 2.59 |  |
|  | serine | 31.81 ± 8.15 | 49.64 ± 15.97 | 19.27 ± 5.15 | 50.96 ± 6.82 |  |
|  | threonine | 25.69 ± 3.85 | 29.81 ± 4.33 | 14.51 ± 4.19 | 21.66 ± 3.57 |  |
|  | tryptophan | 6.00 ± 1.90 | 19.01 ± 5.88 | 3.25 ± 0.91 | 16.17 ± 6.17 |  |
|  | tyrosine | 2.39 ± 0.70 | 4.04 ± 0.45 | 1.60 ± 0.40 | 2.46 ± 0.62 |  |
|  | valine | 11.47 ± 1.53 | 8.95 ± 3.17 | 9.10 ± 1.41 | 12.19 ± 1.56 |  |
| Fatty acids (mg kg^−1^  fresh weight) | alpha-linolenic acid | 316.98 ± 65.89 | 244.15 ± 99.76 | 447.64 ± 117.57 | 403.98 ± 16.51 |  |
|  | arachidic acid | 11.30 ± 1.76 | 6.66 ± 1.58 | 12.50 ± 4.88 | 11.29 ± 1.13 |  |
|  | dodecanoic acid | 83.94 ± 3.59 | 103.92 ± 7.48 | 59.20 ± 11.61 | 95.41 ± 1.04 |  |
|  | linoleic acid | 389.12 ± 57.67 | 307.86 ± 78.29 | 483.04 ± 177.42 | 482.88 ± 8.72 |  |
|  | oleic acid | 158.85 ± 19.73 | 121.70 ± 43.53 | 161.18 ± 107.30 | 205.01 ± 18.01 |  |
|  | palmitic acid | 104.03 ± 9.54 | 85.14 ± 7.43 | 139.81 ± 7.08 | 95.41 ± 1.04 |  |
| Others (mg kg^−1^  fresh weight) | lactic acid | 8.38 ± 2.69 | 9.52 ± 2.23 | 6.49 ± 0.88 | 8.23 ± 2.75 |  |
|  | myo-inositol | 87.04 ± 11.86 | 116.90 ± 33.28 | 90.30 ± 46.65 | 142.36 ± 77.39 |  |
|  | threonic acid | 24.99 ± 4.56 | 13.31 ± 2.00 | 19.75 ± 3.35 | 9.24 ± 1.58 |  |

^1)^BG, big-green stage; ^2)^FR, fully-red stage; SD, standard deviation.
