# Supplementary material for: Comparative transcriptome and metabolome analyses of two strawberry cultivars with different storability
Source: PLoS One. 2020 Dec 2;15(12):e0242556. doi: 10.1371/journal.pone.0242556 (PMC7710044; doi:10.1371/journal.pone.0242556)
Supplement: S14 Table — (DOCX) [file pone.0242556.s021.docx]

**S14 Table. Statistics for analysis of variance for effect of cultivar, stage, and their interaction on selected gene expression**

| **Figure^1)^** | **Source** | **DF** | **Sum of squares** | **Mean squares** | **F** | **Pr > F** |
| --- | --- | --- | --- | --- | --- | --- |
| 5A | Model | 24 | 142.018 | 5.917 | 14.486 | < 0.0001 |
|  | cultivar | 4 | 47.805 | 11.951 | 29.257 | < 0.0001 |
|  | stages | 4 | 70.069 | 17.517 | 42.882 | < 0.0001 |
|  | cultivar × stage | 16 | 24.143 | 1.509 | 3.694 | 0.000 |
|  | Error | 50 | 20.425 | 0.408 |  |  |
|  | Corrected Total | 74 | 162.443 |  |  |  |
| 5B | Model | 24 | 1444.372 | 60.182 | 18.877 | < 0.0001 |
|  | cultivar | 4 | 11.852 | 2.963 | 0.929 | 0.454 |
|  | stage | 4 | 1220.208 | 305.052 | 95.685 | < 0.0001 |
|  | cultivar × stage | 16 | 212.312 | 13.269 | 4.162 | < 0.0001 |
|  | Error | 50 | 159.404 | 3.188 |  |  |
|  | Corrected Total | 74 | 1603.776 |  |  |  |
| 5C | Model | 24 | 74.096 | 3.087 | 8.314 | < 0.0001 |
|  | cultivar | 4 | 1.960 | 0.490 | 1.319 | 0.276 |
|  | stage | 4 | 60.625 | 15.156 | 40.816 | < 0.0001 |
|  | cultivar × stage | 16 | 11.511 | 0.719 | 1.937 | 0.039 |
|  | Error | 50 | 18.566 | 0.371 |  |  |
|  | Corrected Total | 74 | 92.662 |  |  |  |
| 5D | Model | 24 | 4.967 | 0.207 | 17.579 | < 0.0001 |
|  | cultivar | 4 | 0.881 | 0.220 | 18.712 | < 0.0001 |
|  | stage | 4 | 3.241 | 0.810 | 68.811 | < 0.0001 |
|  | cultivar × stage | 16 | 0.845 | 0.053 | 4.488 | < 0.0001 |
|  | Error | 50 | 0.589 | 0.012 |  |  |
|  | Corrected Total | 74 | 5.556 |  |  |  |
| 5E | Model | 24 | 9.091 | 0.379 | 11.464 | < 0.0001 |
|  | cultivar | 4 | 0.928 | 0.232 | 7.018 | 0.000 |
|  | stage | 4 | 5.645 | 1.411 | 42.711 | < 0.0001 |
|  | cultivar × stage | 16 | 2.518 | 0.157 | 4.763 | < 0.0001 |
|  | Error | 50 | 1.652 | 0.033 |  |  |
|  | Corrected Total | 74 | 10.743 |  |  |  |
| 5F | Model | 24 | 7.651 | 0.319 | 8.167 | < 0.0001 |
|  | cultivar | 4 | 0.741 | 0.185 | 4.744 | 0.003 |
|  | stage | 4 | 5.875 | 1.469 | 37.625 | < 0.0001 |
|  | cultivar × stage | 16 | 1.035 | 0.065 | 1.658 | 0.088 |
|  | Error | 50 | 1.952 | 0.039 |  |  |
|  | Corrected Total | 74 | 9.602 |  |  |  |
| 5G | Model | 24 | 8.521 | 0.355 | 8.970 | < 0.0001 |
|  | cultivar | 4 | 1.847 | 0.462 | 11.665 | < 0.0001 |
|  | stage | 4 | 5.507 | 1.377 | 34.781 | < 0.0001 |
|  | cultivar × stage | 16 | 1.167 | 0.073 | 1.843 | 0.051 |
|  | Error | 50 | 1.979 | 0.040 |  |  |
|  | Corrected Total | 74 | 10.500 |  |  |  |
| 5H | Model | 24 | 12.768 | 0.532 | 7.520 | < 0.0001 |
|  | cultivar | 4 | 0.874 | 0.218 | 3.088 | 0.024 |
|  | stage | 4 | 9.625 | 2.406 | 34.014 | < 0.0001 |
|  | cultivar × stage | 16 | 2.269 | 0.142 | 2.005 | 0.031 |
|  | Error | 50 | 3.537 | 0.071 |  |  |
|  | Corrected Total | 74 | 16.305 |  |  |  |
| 5I | Model | 24 | 17.795 | 0.741 | 9.869 | < 0.0001 |
|  | cultivar | 4 | 4.803 | 1.201 | 15.982 | < 0.0001 |
|  | stage | 4 | 10.429 | 2.607 | 34.701 | < 0.0001 |
|  | cultivar × stage | 16 | 2.563 | 0.160 | 2.132 | 0.021 |
|  | Error | 50 | 3.757 | 0.075 |  |  |
|  | Corrected Total | 74 | 21.551 |  |  |  |
| 5J | Model | 24 | 29.553 | 1.231 | 7.299 | < 0.0001 |
|  | cultivar | 4 | 10.711 | 2.678 | 15.872 | < 0.0001 |
|  | stage | 4 | 13.260 | 3.315 | 19.650 | < 0.0001 |
|  | cultivar × stage | 16 | 5.583 | 0.349 | 2.068 | 0.026 |
|  | Error | 50 | 8.435 | 0.169 |  |  |
|  | Corrected Total | 74 | 37.989 |  |  |  |
| S7A | Model | 24 | 5.176 | 0.216 | 5.092 | < 0.0001 |
|  | cultivar | 4 | 0.305 | 0.076 | 1.802 | 0.143 |
|  | stage | 4 | 3.654 | 0.913 | 21.566 | < 0.0001 |
|  | cultivar × stage | 16 | 1.217 | 0.076 | 1.796 | 0.059 |
|  | Error | 50 | 2.118 | 0.042 |  |  |
|  | Corrected Total | 74 | 7.294 |  |  |  |
| S7B | Model | 24 | 6.904 | 0.288 | 7.093 | < 0.0001 |
|  | cultivar | 4 | 1.459 | 0.365 | 8.993 | < 0.0001 |
|  | stage | 4 | 3.110 | 0.777 | 19.168 | < 0.0001 |
|  | cultivar × stage | 16 | 2.335 | 0.146 | 3.599 | 0.000 |
|  | Error | 50 | 2.028 | 0.041 |  |  |
|  | Corrected Total | 74 | 8.932 |  |  |  |

^1)^ Data for each figure was used for two-way ANOVA; DF, degree of freedom; F, F value; Pr > F, the significance probability value associated with the F value.
